# Pharmacologically induced reversible hypometabolic state mitigates radiation induced lethality in mice

**DOI:** 10.1038/s41598-017-15002-7

**Published:** 2017-11-02

**Authors:** Subhajit Ghosh, Namita Indracanti, Jayadev Joshi, Jharna Ray, Prem Kumar Indraganti

**Affiliations:** 1 Division of Radiation Biosciences, Institute of Nuclear Medicine and Allied Sciences, Brig SK Majumdar Road, Timarpur, Delhi India; 2 S. N. Pradhan Centre for Neuroscience, University of Calcutta, Kolkata, India

## Abstract

Therapeutic hypothermia has proven benefits in critical care of a number of diseased states, where inflammation and oxidative stress are the key players. Here, we report that adenosine monophosphate (AMP) triggered hypometabolic state (HMS), 1–3 hours after lethal total body irradiation (TBI) for a duration of 6 hours, rescue mice from radiation-induced lethality and this effect is mediated by the persistent hypothermia. Studies with caffeine and ^6^N-cyclohexyladenosine, a non-selective antagonist and a selective agonist of adenosine A1 receptor (A1AR) respectively, indicated the involvement of adenosine receptor (AR) signaling. Intracerebroventricular injection of AMP also suggested possible involvement of central activation of AR signaling. AMP, induced HMS in a strain and age independent fashion and did not affect the behavioural and reproductive capacities. AMP induced HMS, mitigated radiation-induced oxidative DNA damage and loss of HSPCs. The increase in IL-6 and IL-10 levels and a shift towards anti-inflammatory milieu during the first 3–4 hours seems to be responsible for the augmented survival of HSPCs. The syngeneic bone marrow transplantation (BMT) studies further supported the role of radiation-induced inflammation in loss of bone marrow cellularity after TBI. We also showed that the clinically plausible mild hypothermia effectively mitigates TBI induced lethality in mice.

## Introduction

Hibernating animals survive extreme environmental settings, including anoxia, freezing temperatures by suppressing their metabolic rate (<5% of resting rate), reducing core body temperature (T_c_) (falling to near ambient) and switching to anaerobic metabolism^1–3^. Non-hibernating mammals can barely survive a few tens of minutes without oxygen or reduced T_c_^4^. The extreme ability of hibernators to survive otherwise lethal conditions generated significant interest, for obvious implications, in emergency medicine^5^. In 2008, Blackstone *et al*. have demonstrated that hydrogen sulfide (H_2_S), a small gaseous molecule, can trigger a deep HMS in laboratory mouse a naturally non-hibernating animal which later on showed to be protective against a variety of stressors^6–10^. However, H_2_S failed to render similar effect in larger naturally non-hibernating mammals thereby raising concerns about its translational success^11^. In fact the requirement for higher concentrations and the inability of H_2_S to inhibit the mitochondrial complex IV due to small window of opportunity has been proposed to be some of the reasons for the failure in large mammals^12^. Nevertheless, amongst different molecules which have been identified to be effective HMS inducers, adenosine monophosphate (AMP) can be considered important. Initially in 2006, Zhang *et al*. have identified 5′-AMP, a natural metabolite and an approved neutraceutical, as a key mediator of torpor like state in mice^13^. Since then AMP has been intensively investigated as a pharmacological means for induction of hibernation like state in natural non-hibernators and is known to protect model organisms from a number of shocks including oxidative stress^14–16^. Interestingly, the synthetic torpor has also been proposed to have implications in the management of cancer^17^. During HMS, tissues tolerate higher radiation doses and it has direct implications in radiotherapy of cancers as higher doses of radiation can be targeted to tumors which normally kill an awake and non-hibernating cancer patient. Apart from cancer patients, HMS has also been proposed to be a promising tool for deep space travel. As it ensures low metabolic consumption, it may lead to reduced requirement for food, liquid and life support systems which have direct consequences on payload which in turn critically determines long space flights^18,19^. However, practical application of HMS in deep space travel is still a hypothetical area and may take decades before it can be realized. Nevertheless, synthetically induced HMS can have significant immediate medical applications, including radiation protection^17^.

Free radicals generated by radiolysis of water mediate a massive loss of hematopoietic and gastrointestinal tract cells through apoptosis and have been associated with acute radiation syndromes in animals and humans^20–22^. In this current global scenario, with several small and large states acquiring nuclear power and stockpiling weapons of mass destruction, the threat perception has increased more than ever^23^ and there is an urgency for the development of countermeasures against nuclear exigencies^24,25^. A number of molecules with diverse functions have been reported to rescue both small and large mammals from lethal effects of ionizing radiation^26^. However, there are no radioprotectors or mitigators available, as of now, which are safe and useful for humans during nuclear exigencies^26^. Classically, direct cytotoxicity by radiation has been identified as the major causative pathway leading to cell, tissue and organism death. However, more recently loco regional as well as systemic inflammation have also been implicated in dysfunction of bone marrow and other organs^21,22^. In fact, both experimental and clinical experiences have suggested the importance of developing countermeasures in the context of systemic radiation injury rather than targeting normal tissues of individual organs^27^. In homeotherms the cellular and tissue stress responses, post-irradiation, is governed by metabolic activity which in turn depends upon body temperature. Low temperature renders radio-resistance and this temperature effect was demonstrated in a variety of model systems including naturally hibernating dormice and ground squirrel^28–32^. However, most of the studies demonstrating the radioprotective effect of hypothermia or hibernation per se were done in cellular systems or with naturally hibernating mammals thereby making it difficult to extrapolate the effect to non-hibernating mammals. Moreover, the protective effect of HMS when induced after TBI has not been demonstrated.

We reason that the transient slowing down of metabolism after TBI would subside the radiation induced early inflammatory responses and reduce the loss of bone marrow stem cells. Indeed, in this study, we demonstrated that pharmacologically (using AMP) triggered HMS modulate radiation induced early inflammatory response and rescued both long- and short-term hematopoietic stem cells (HSCs) in the bone marrow leading to effective hematopoietic recovery and increased survival.

## Materials and Methods

### Mouse Experiments

Animal handling and experiments with mice were carried out in accordance with the approval from the Committee on the Ethics of Animal Experiments, Institute of Nuclear Medicine and Allied Sciences (INMAS), Defence Research and Development Organization (DRDO), Delhi, India (Institutional Ethical committee number under which this study has been approved is INM/IAEC/2013/03/04 (Protocol no: TD-10018; GO/a/99/CPCSEA). Male and female mice (C57BL/6J, BALB/c) of 8–10 weeks of age (25 ± 2 g), were used for this study. For some experiments, 8–10 weeks old female Sprague Dawley rats weighing 180 ± 10 g were used. The animals were kept at Experimental Animal Facility, INMAS, Delhi, India at an ambient temperature (T_a_) and relative humidity of 23–25 °C and 55% respectively. Unlimited mouse chow (Golden Feeds, Delhi, India) and tap water was provided ad libitum, and the animals were maintained on a 12 hours light and 12 hours dark cycle. All the efforts were made to minimize animal number and suffering. All experiments were performed following the protocols approved by the Committee on the Ethics of Animal Experiments of INMAS, Delhi, India.

### Radiation exposure, treatments and survival study

TBI was performed using a ^60^Co-γ source irradiator (Bhabatron-II tele-therapy unit, Panacea Medical Technologies, Bengaluru, India). For radio-mitigation studies, mice were exposed to 8Gy (C57BL/6J hereafter referred to as B6) or 7Gy (BALB/c) at a dose rate of 1.00Gy/minute on a rotating chamber and the irradiation field size was 35 × 35 cm^2^. Animals were partially restrained and then exposed to irradiation in a group of 4. All irradiations were consistently carried out between 12:00 to 01:00 p.m. to minimize the chronosensitivity^33^. The mapping of exposure rates was carried out by an Atomic Energy Regulatory Board (AERB) authorized health physicist using a calibrated RadCal 0.6 cm^3^ therapy grade ion chamber and electrometer system. One hour after TBI, mice were either treated with vehicle (phosphate buffered saline) or AMP (0.5 mg/g b.w.; Sigma-Aldrich, St. Louis, MO, USA) via intraperitoneal route (i.p.). In some instances, amifostine (214 mg/kg b.w.; a prototype pharmacologic radioprotector that functions via free radical scavenging)^34^ was administered i.p., 8 hours after TBI and AMP treatment. For evaluating the radiomitigative effect of adenosine A1 receptor (A1AR) agonist,^6^N-cyclohexyladenosine (CHA; 1 mg/kg b.w.)^35^. Animal’s overall health, appearance, body weight and mortality were recorded daily till the end of the experimentation (till 30 post-irradiation days) and the survival was compared over the first 30 days between each of the treated groups and the radiation-only control group. The overall health of non-irradiated mice which were administered with either AMP (0.5 mg/g b.w.) or CHA (1 mg/kg b.w.) was free from adverse side effects over the period of study (30 days). After different treatments, animals were housed and maintained at experimental animal facility without any supportive care. Animals showing a combined score of more than 8 (posture score, eye appearance score and activity score) were humanely euthanized to minimize the pain and distress^36^.

### Induction of HMS in mice

HMS was induced using AMP by essentially following the approach reported by Daniels *et al*.^37^. HMS was induced by administering 0.5 mg/g b.w. of AMP (prepared in phosphate-buffered saline) through i.p. route. The animals were then kept at room temperature (25 °C) or at a T_a_ of 15 °C (for maintaining HMS) for a total duration of 6 hours before they were brought back to room temperature for recovery. The mice which did not respond to the treatment and were awake during the incubation at 15 °C were removed from the study. To evaluate the role of AR in AMP mediated HMS, caffeine (40 mg/kg b.w.) was administered (i.p.) 30 minutes prior to the administration of AMP. Detailed methodology for measurement of T_c_, surface temperature (T_s_), peripheral blood flow, activity profile, tissue ATP levels, blood glucose, pyruvate and lactate levels, hif1-α expression, haematological profiles, and spatial dynamics of blood components during the course of HMS is presented in supplementary information. Bone marrow cell count, cytologic evaluation, HSPCs enumeration, alkaline comet assay, cytokine measurements in serum and syngeneic BMT was also done to assess the effect of HMS on radiation induced hematopoietic form of acute radiation syndrome (hARS) (for detailed methodology see supplementary information).

### Animals, ICV Injection of AMP and measurement of T_c_

For establishing the role of central activation of the AR in induction of hypometabolic state by AMP, direct intracerebroventricular (i.c.v) injection of AMP and T_b_ measurements were done (for detailed methodology see supplementary information).

### Statistical analysis

All the results are presented as mean ± standard error of the mean (SEM). All statistical analysis were performed with GraphPad Prism from GraphPad Software (v5.0, La Jolla, CA, USA). Two way ANOVA repeated measure followed by boneferoni post-test was used for multiple comparisons and Student’s t-test was used for single comparison. The kinetics of overall survival, defined as time from the date of irradiation to the date of death for the mice under study, was analysed using log-rank test (two-tailed). Differences with a p-value less than 0.05 were considered statistically significant.

### Data availability

All relevant data are contained within the article and Supplementary files, or are available from the authors on request.

## Results

### HMS in un-irradiated and lethally irradiated mice through AR

**Figure 1:**
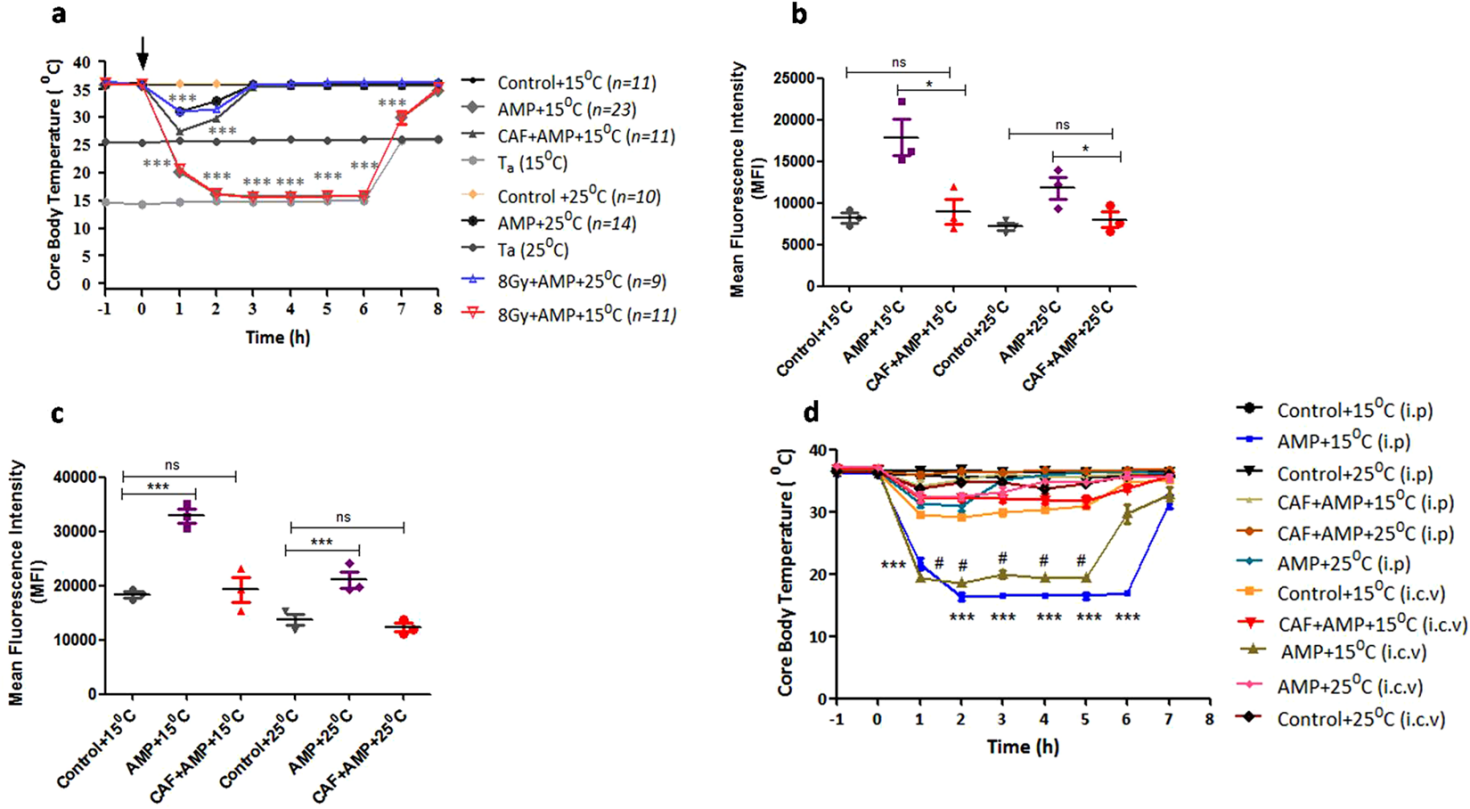
Effect of AMP induced HMS on Tc. (**a**) B6 mice were administered intraperitoneally 0.5 mg/g b.w. of AMP with or without indicated treatments and immediately transferred to a T_a_ of 15 °C for a period of 6 hours. Thereafter, mice were brought back to room temperature (25 °C) and allowed to recover. The Tc was monitored on an hourly basis using RET3 rectal probe (two way ANOVA after Boneferoni post-test: F = 384.7and P < 0.0001 for interaction, F = 692.5 and P < 0.0001 for time, F = 1629.0 and P < 0.0001 for treatment, ***p < 0.001 for all AMP treated group at 150 C (1–6 h) and at 260 C along with CAF (1–2 h) when compared to control + 15 °C). Changes in the expression of Hif1α in frontal cortex (**b**) and femur (**c**) 6 hours after indicated treatments. (**d**). Central activation of adenosine receptor in AMP mediated HMS. Vehicle or AMP in the presence or absence of caffeine was administered directly into frontal cortex (intracerebroventricular region) and changes in Tc was measured on an hourly basis for the duration of the experiment. Each value is a mean ± SEM (n = 3–6 mice/group) and comparisons were made between indicated groups. *p < 0.05, **p < 0.01, ***p < 0.001, ns not significant. Caf represents caffeine.

Mice that were given vehicle alone displayed no change in T_c_ regardless of whether T_a_ was 25 or 15 °C (Fig. 1a). In contrast, mice that received AMP and placed at T_a_ of 25 °C (hereafter referred as AMP (+T_a_ 25 °C) displayed a decrease in T_c_ within 15 minutes which gradually reduced to a minimum value (~30 °C) reached after 1 hour. Thereafter, the T_c_ then gradually increased and reached to normal levels by 3 hours. However, mice that were treated with AMP (+T_a_ 15 °C) exhibited a steep decrease in T_c_ and by 2 hours the T_c_ plummeted to near T_a_ (~16 °C). The T_c_ remained at near ambient temperature until the animals were shifted from T_a_ of 15 °C to T_a_ of 25 °C and within 2 hours of being at 25 °C the T_c_ returned to normal levels (~36 °C). In about 1 hour after the administration of AMP (+T_a_ 15 °C) the animals were in a state of deep sleep without any movement except occasional involuntary movements (Supplementary video). Unlike AMP (+T_a_ 15 °C) treated animals, the untreated mice placed at a T_a_ of 15 °C were found to be active except that they were cuddling, to minimize the heat loss. Mice exposed to TBI and treated with AMP also responded in a similar fashion and the reduction and recovery kinetics of T_c_ was found to be similar to that of un-irradiated control mice treated with AMP (+T_a_ 15 °C) (Fig. 1a). To verify the involvement of AR in AMP mediated induction of HMS in case of lethally irradiated mice the effect of caffeine, a pan-AR antagonist, was investigated. Pre-treatment of mice with caffeine (40 mg/kg b.w.) 30 minutes prior to AMP (+T_a_ 15 °C) treatment almost completely abolished the induction of HMS. In caffeine treated animals after 1 hour of AMP administration the T_c_ reached to a minimum (~28 °C) level which thereafter recovered to normal levels by 3 hours. Similarly, AMP mediated HMS lead to induction of hypoxia in both brain (Fig. 1B) and femur (Fig. 1c) which was abrogated by pre-treatment of caffeine (see supplementary information for detailed results).

AMP has been previously reported to induce a reversible HMS in mice through AR^37–40^. Downstream activators of oxidative stress and electromagnetic pulse are known to regulate the expression of AR^41,42^. A key question raised from these studies was whether AMP can induce reversible HMS in lethally irradiated mice akin to normal un-irradiated mice. Mice exposed to TBI and treated with AMP exhibited a reduction and recovery kinetics of T_c_ similar to that of un-irradiated control mice treated with AMP (+T_a_ 15 °C) and found to be mediated through AR signalling (Fig. 1a) (for detailed results see supplementary information and Supplementary Figs 1–9). In order to confirm the involvement of central activation of adenosine receptor mediated signalling, AMP was administered directly into intracerebroventricular (ICV) part of the brain and T_a_ was monitored over a period of 8 hours (Fig. 1d). Animals which received AMP in the ICV region have shown a significant reduction in the T_c_ and the kinetics of induction and recovery was found to be similar to that observed upon intra peritoneal administration (Fig. 1d).

### HMS effectively mitigated TBI induced lethality in mice

**Figure 2:**
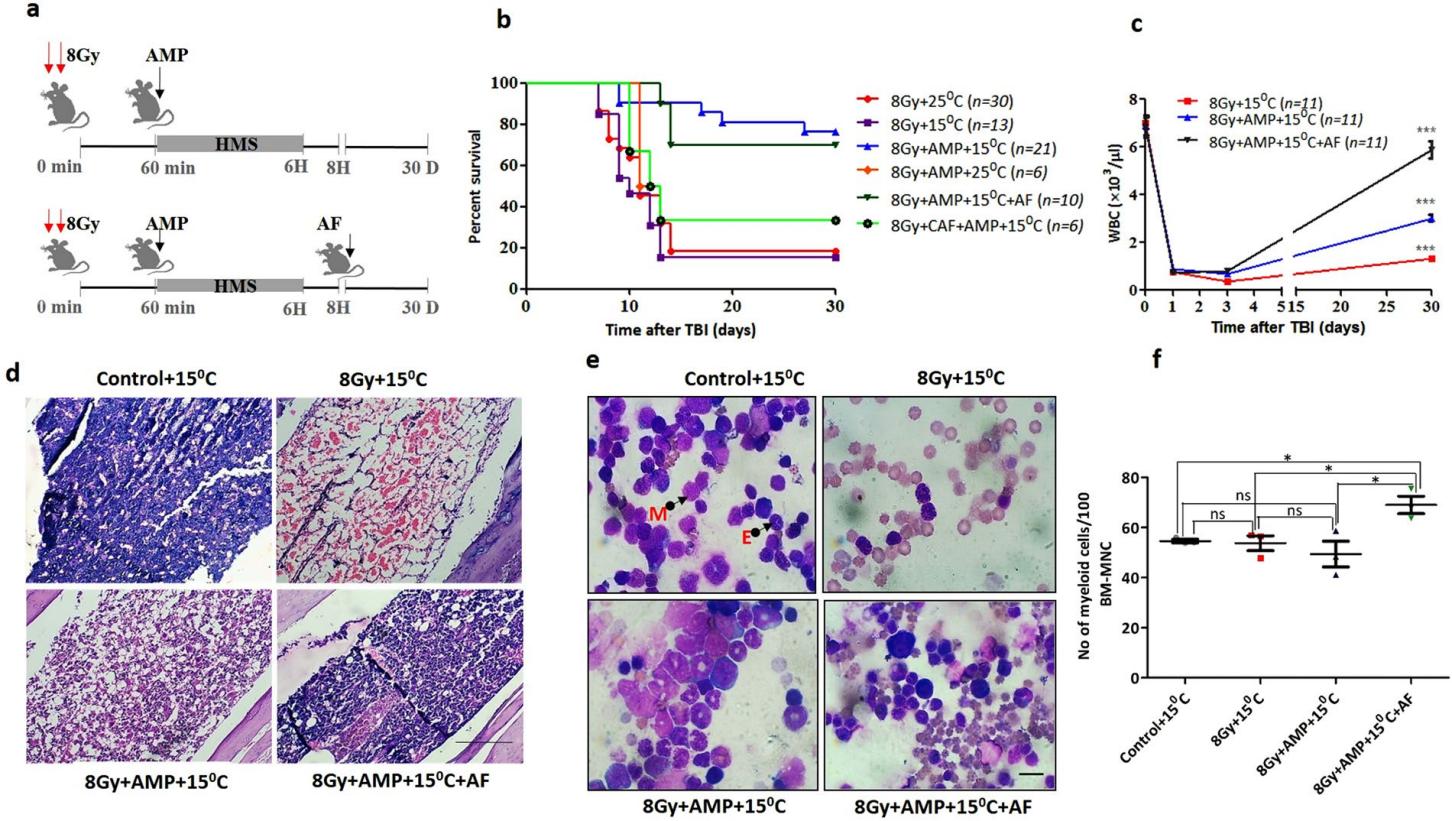
AMP + 15 °C induced HMS mitigates lethal effects of TBI in mice. (**a**) The schema of experimental setup. B6 male mice were exposed to 8Gy of ^60^Co-γ rays and 1 hour later either vehicle or AMP (0.5 mg/g b.w.) was administered through intraperitoneal route followed by placing them at 15 °C or room temperature for 6 hours. In some experiments after the animals were recovered from HMS, amifostine was administered through intraperitoneal route. (**b**) Kaplan-Meier plots of 30 days survival of mice. (**c**) Changes in absolute WBC counts over time in B6 mice exposed to lethal doses of gamma radiation and treated with AMP + 15 °C alone or with amifostine (two way ANOVA after Boneferoni post-test: F = 1.963 and P = 0.0793 for interaction, F = 180.00 and P < 0.0001 for time, F = 1.78 and P = 0.01 for treatment, ***p < 0.001 and *p < 0.05 when compared to 8Gy alone). (**d**) Representative images of longitudinal section of femur harvested 30 days after different treatment and stained with H&E. (**e**) Representative images of bone marrow smears prepared 24 hours after different treatments and stained with MayGrunwald-Giemsa. M, E represents myeloid and erythroid series respectively, scale bar = 20 µm (**f**) Changes in the number of myeloid progenitors per 100 nucleated bone marrow cells. Each value is a mean ± SEM (n = 4–6 mice/group) and comparisons were made between indicated groups. *p < 0.05, **p < 0.01, ***p < 0.001. Caf and AF represents caffeine and amifostine respectively.

The present study aimed at understanding the mitigative effect of HMS against radiation induced hematopoietic form of acute radiation syndrome and death (hARS). The radiation doses selected for this study, 8Gy is known to effectively induce hARS and yield around 85–90% thereby enabling survival of a few animals (15%) which is critically required for monitoring the changes in irradiation alone group and for comparing the mitigative effect of irradiated animals treated with AMP. B6 male mice exposed to TBI (8Gy) followed by placing them at T_a_ of 15 °C for 6 hours did not show any deviation from TBI induced mortality in animals kept at T_a_ of 25 °C and resulted in ~15% survival by 30 days with a mean survival time of 12 ± 2 days (Fig. 2a,b). In contrast, HMS (AMP + T_a_ 15 °C) induced 1 hour after TBI (hereafter referred to as TBI + HMS group) resulted in a significant increase in the survival advantage (~60%) over vehicle treated TBI (+T_a_15 °C) (hereafter referred to as TBI group) by day 30 (Fig. 2b). Administration of AMP at different time intervals after TBI indicated that it was effective when induced within 3 hours after TBI (data not shown). To test whether inclusion of a known radioprotector offers any additive effect on overall survival, the effect of amifostine (hereafter referred to as AF) administered after 8 hours of AMP treatment was investigated (Fig. 2b). Although, this treatment did not improve the survival against TBI over the HMS treatment group but it resulted in an improved general appearance of animals (appeared healthier and active). Similarly, the TBI + HMS or TBI + HMS + AF groups exhibited better recovery from TBI induced loss of body weight (for detailed results see supplementary information and Supplementary Fig. 9a).

### HMS improves recovery from radiation induced leukocytopenia, erythropenia and restores spleen weight

Irrespective of T_a_, TBI caused a massive loss of WBC (>88%), lymphocytes (90%) and granulocytes (87%) 24 hours after TBI when compared to vehicle treated un-irradiated (+T_a_15 °C) control group (hereafter referred to as control), (Fig. 2c). On day 3 the number of WBCs were reduced while on day 30 the surviving animals showed a moderate recovery (18.5%). HMS did not affect the radiation induced loss of WBCs and their number after 24 and 72 hours were similar to that of the vehicle treated TBI group. However, the animals showed a better recovery post day 3 and the number of WBCs on day 30 was 2.85 × 10^3^ cells/µl (39% recovery). AF, which was administered 8 hours after induction of HMS in TBI animals also failed to prevent the radiation induced loss of WBC but significantly enhanced their reclamation on day 30 (70% recovery) when compared to TBI (p = 0.001) or TBI + HMS groups (p = 0.001). Correspondingly, both the lymphocytes and granulocytes, have shown a response and recovery similar to that of WBCs (Supplementary information and Supplementary Fig. 9b,c). Similarly, HMS or HMS + AF protected RBC (Supplementary Fig. 9d) and spleen (Supplementary Fig. 9e) from radiation induced changes (Supplementary information).

### HMS prevents radiation induced loss of HSPCs in bone marrow

Evaluation of histopathological changes, on day 30, in longitudinally sectioned and H&E (Haemotoxylin and Eosin) stained femurs showed that, TBI caused aplasia with few islands of nucleated hematopoietic cells and massive deposition of adipose cells (Fig. 2d). HMS significantly improved recovery from radiation induced aplasia and adipogenesis. AF treatment further enhanced bone marrow recovery corroborating with the recovery status of blood cells in the peripheral circulation on day 30. As recovery from radiation induced hARS depends upon the number of surviving and functional HSCs in bone marrow, the cellular status was assessed. Bone marrow smears prepared 24 hours later from TBI mice showed that radiation almost completely sterilized the bone marrow with few pockets of nucleated hematopoietic cells (Fig. 2e). However, the frequency of myeloid cells in irradiated animals did not change when compared to untreated control group (p = 0.79) albeit more number of microscopic fields were counted when compared to control group (Fig. 2f). HMS also did not influence the radiation induced change in the frequency of myeloid cells when compared to control (p = 0.367) or TBI animals (p = 0.357). However, HMS + AF treatments resulted in a significant escalation in the frequency of myeloid series when compared to control (p = 0.014) or TBI groups (p = 0.028) (Fig. 2f). This increases in TBI + HMS + AF group, was in line with a myeloid bias observed in the peripheral blood counts on day 30. Similarly, TBI or TBI + HMS did not influence the frequency of erythroid series when compared to control group (Supplementary Fig. 9f). However, TBI + HMS + AF treatments resulted in a significant reduction in the frequency of erythroid series when compared with control (p = 0.0021) or TBI groups (p = 0.0002).

**Figure 3:**
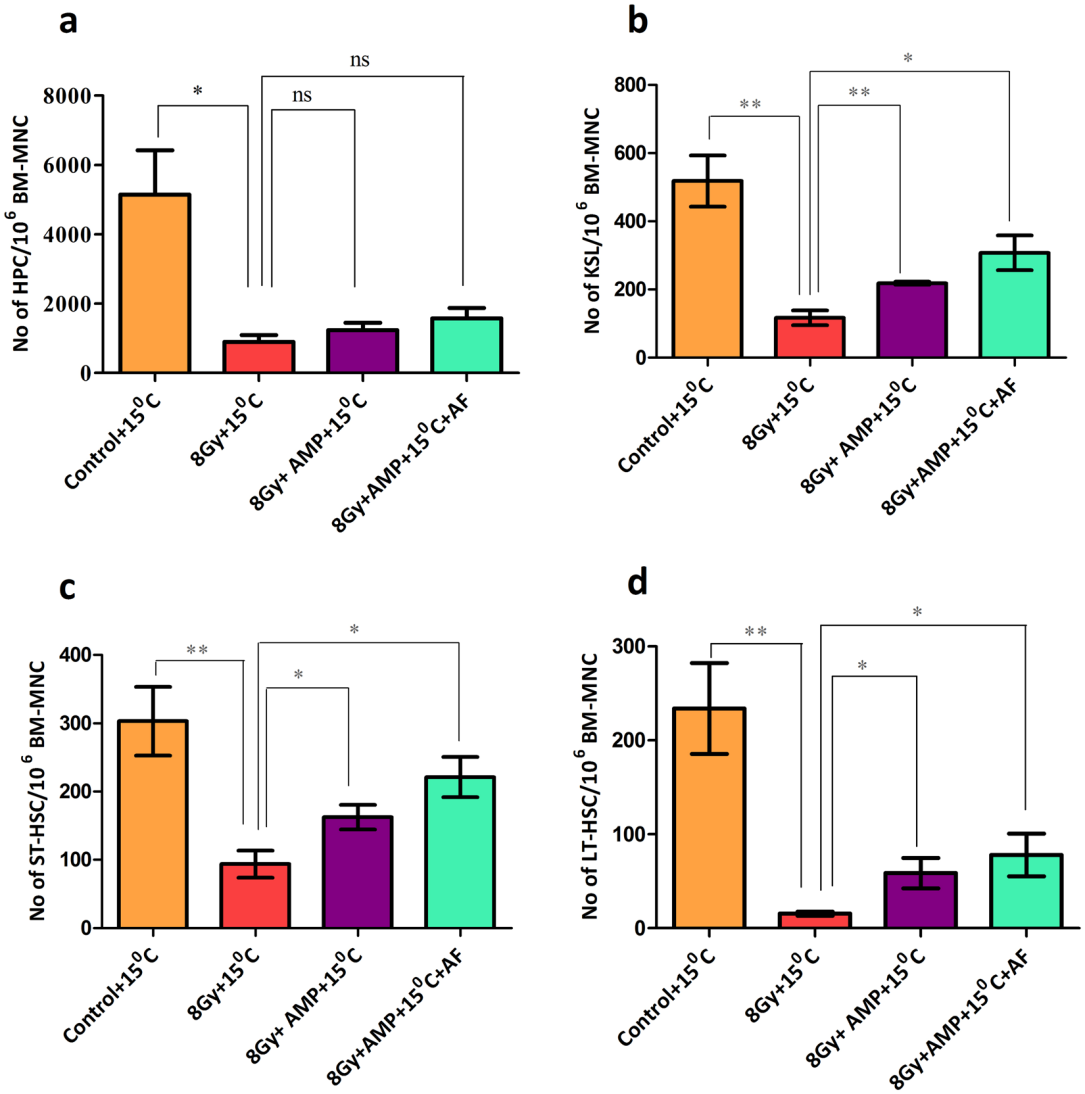
AMP + 15 °C induced HMS protects both short and long term repopulating cells but not multipotent progenitors. B6 male mice were exposed to lethal dose (8 Gy) of gamma radiation followed by intraperitoneal administration of AMP and placed at 15 °C for 6 hours. For amifostine treatment, after 6 hours of AMP + 15 °C animals were allowed to recover at room temperature for 2 hours and then amifostine (214 mg/kg bw, i.p.) was administered. 24 hours after different treatments, bone marrow was harvested and processed, stained and different HSPCs were enumerated. The numbers of HPCs (**a**) KSL (**b**) ST-HSCs (**c**) and LT-HSCs (**d**) in the bone marrow harvested 24 hours after different treatments and presented as number of cells per million BMMNCs. Each value is a mean ± SEM (n = 6 animals/group) and comparisons were made between indicated groups. *p < 0.05, **p < 0.01, ***p < 0.001, ns not significant. AF represents amifostine.

Then to identify the subsets of HSPCs which were rescued from radiation induced loss by HMS, flow cytometric approach was used (Fig. 3a–d, Supplementary Fig. 10a). TBI caused a significant loss (18% surviving when compared to control (100%) of highly proliferating hematopoietic progenitor cells (HPCs; Lin^−^Sca1^−^c-kit^+^ cells) in vehicle treated animals after 24 hours. HMS, although statistically not significant when compared to TBI group, apparently countered radiation induced depletion of HPCs and the surviving fraction was found to be 24% (without AF; p = 0.294) and 30% (with AF; p = 0.107) after 24 hours (Fig. 3a). Similarly, TBI depleted the KSL (Lin^−^Sca1^+^c-Kit^+^ cells), a fraction of BM which contains the HSCs, in vehicle treated animals within 24 hours to 17% (Fig. 3b). Unlike with HPCs, HMS significantly protected KSL fraction (42%; p = 0.003) from radiation induced depletion (Fig. 3a,b). AF reinforced the radiomitigative effect of HMS 59% (p = 0.013 when compared to TBI group). When compared to control group, only 30 and 6% of ST-HSCs (Lin^−^Sca1^+^c-Kit1^+^CD34^+^ cells), and LT-HSCs (Lin^−^Sca1^+^c-Kit1^+^CD34^−^ cells) were found to be surviving after 24 hours, in TBI animals. HMS rescued both ST-HSCs (46%; p = 0.04) and LT-HSCs (53%; p = 0.037) from radiation induced depletion. AF reinforced the radiomitigative effect of HMS and the surviving ST-HSCs and LT-HSCs were found to be 72% (p = 0.011) and 66% (p = 0.033) respectively (Fig. 3c,d).

### HMS effectively prevented TBI induced loss of mitochondrial membrane potential (MMP), DNA damage and apoptosis in ST and LT-HSCs cells but not in HPCs

**Figure 4:**
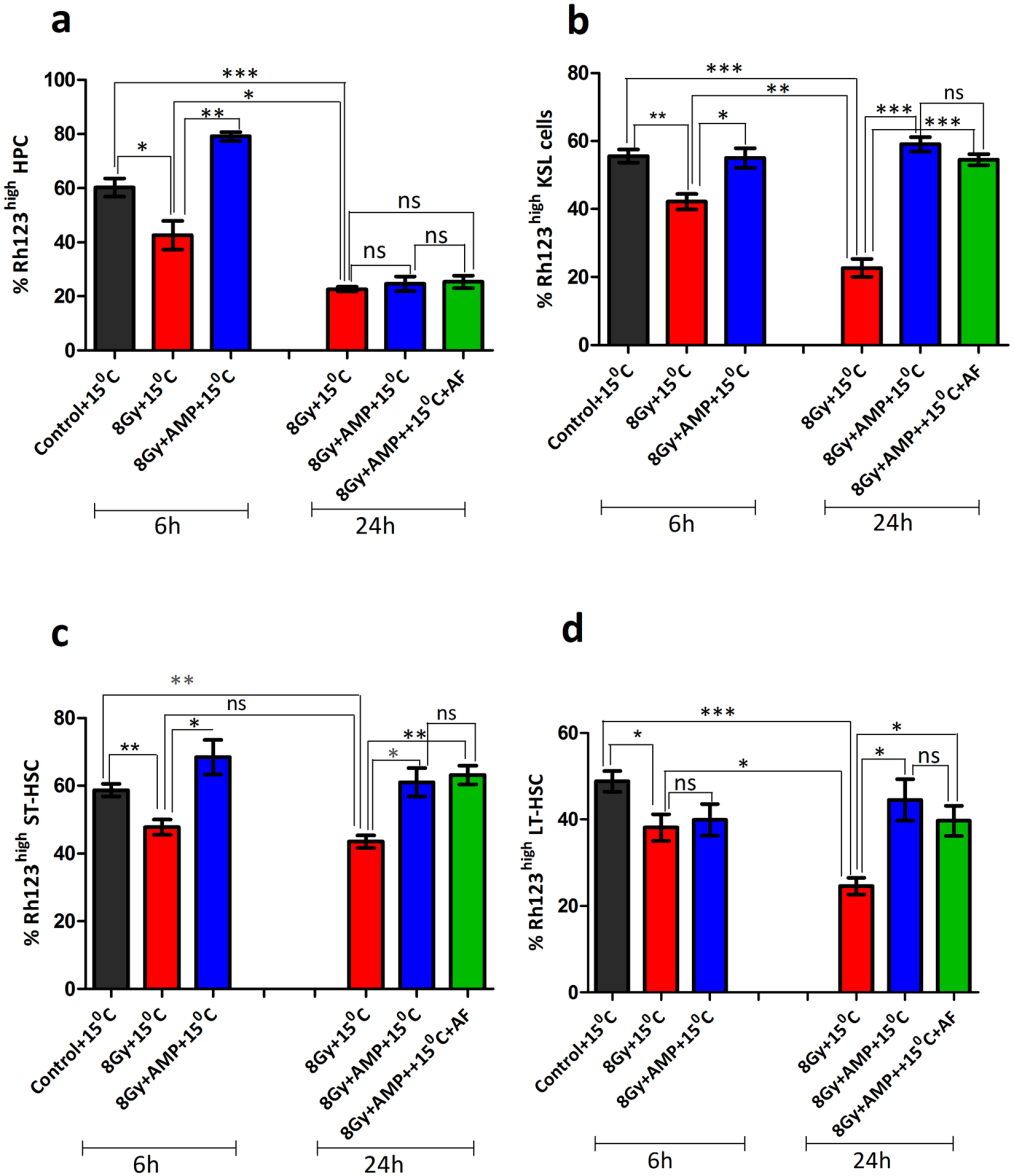
AMP induced HMS prevents radiation induced reduction in MMP in bone marrow stem and progenitor population. Frequencies of highly positive MMP cells in different stem and progenitor population 6 or 24 hours different treatments. The fraction of Rh123 high HPCs (**a**) KSL (**b**) ST-HSCs (**c**) LT-HSCs (**d**) are presented as % of Rh123 high population. In case of LT-HSCs a minimum of 50 cells were used for calculating changes in MMP. Each value is a mean ± SEM (n = 6 animals/group) and comparisons were made between indicated groups. *P < 0.05, **p < 0.01, ***p < 0.001, ns not significant. AF represents amifostine.

**Figure 5:**
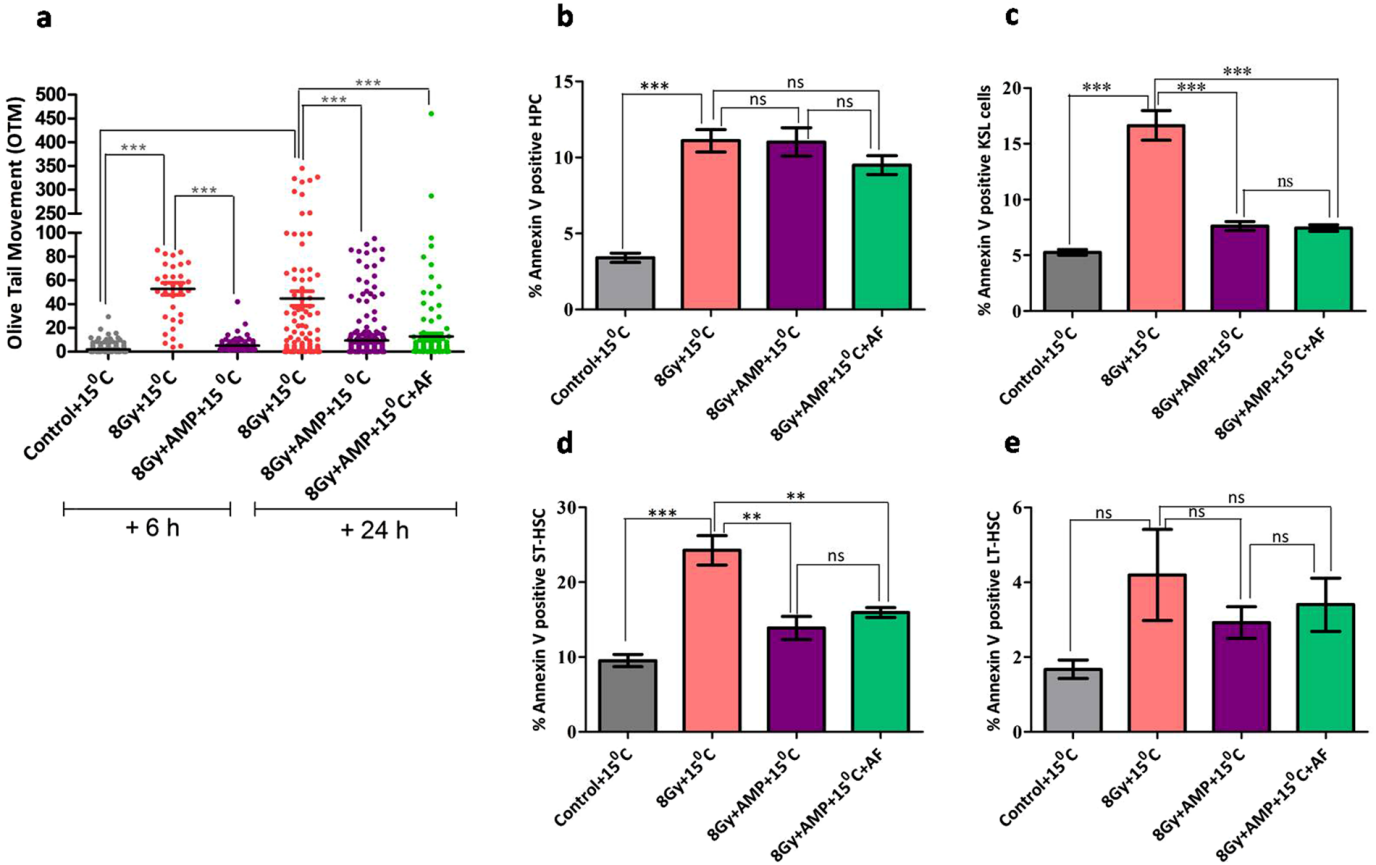
AMP induced HMS mitigates radiation induced DNA damage in Lin^−^ population of bone marrow and rescues different subsets of HSPCs from cell death. (**a**) DNA damage was measured as Olive tail moment in Lin^−^ fraction of bone marrow collected 6 or 24 hours after of TBI with or without different treatments. (**b**) The cells undergoing apoptosis were determined as annexin V^+^ fraction and presented as percent of cells undergoing apoptosis. Each value is a mean ± SEM (n = 6 animals/group) and comparisons were made between indicated groups. *p < 0.05, **p < 0.01, ***p < 0.001, ns not significant. AF represents amifostine.

**Figure 6:**
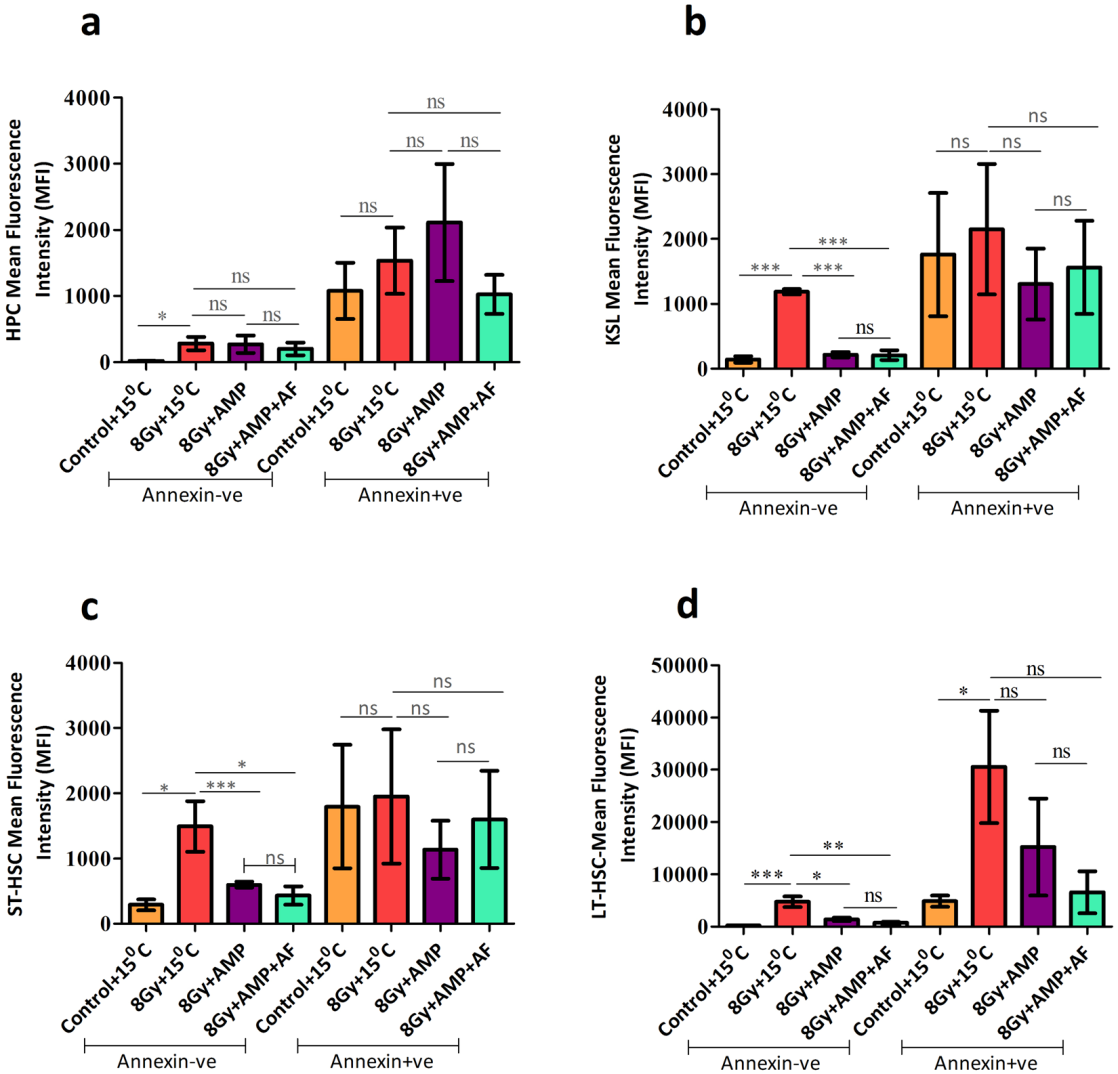
AMP induced HMS mitigates radiation changes in cell surface c-Kit expression in annexin V^−^ but not in annexin V^+^ fractions of different subsets of HSPCs. B6 male mice were exposed to lethal dose (8 Gy) of gamma radiation followed by intraperitoneal administration of AMP and placed at 15 °C for 6 hours. For amifostine (AF) treatment, after 6 hours of AMP + 15 °C animals were allowed to recover at room temperature for 2 hours and thereafter amifostine (214 mg/kg b.w., i.p.) was administered. 24 hours after different treatments, bone marrow was harvested, processed, stained and different HSPCs and surface c-Kit expression was determined as mean fluorescent Intensity (MFI) in annexin V^−^ and annexin V^+^ fractions of HPCs (**a**), KSL (**b**), ST-HSCs (**c**) and LT-HSCs (**d**) was determined flow cytometrically. Each value is a mean ± SEM (n = 6 animals/group) and comparisons were made between indicated groups. *p < 0.05, **p < 0.01, ***p < 0.001, ns not significant. AF represents amifostine.

As the enumeration studies fail to predict the functional competency of surviving HSPCs, the functional status as, changes in MMP, DNA damage and apoptosis, was investigated in the surviving HSPCs at 6 and 24 hours after treatment (Figs 4,5 and 6). We used rhodamine 123 (Rh123), a dye whose accumulation is dependent upon the polarization status of the membranes^43^ and previously used by others for specification of haematopoietic stem cell fate^44^, for assessing the metabolic status of different HSPCs. Bone marrow of control animals have shown that 60, 58, 67 or 47% of HPC, KSL, ST-HSCs and LT-HSCs possessed highly polarized mitochondria (Rh123^high^) (Fig. 4a–d, Supplementary Fig. 10). TBI significantly lowered the MMP in all four subsets of HSPCs in vehicle treated animals both after 6 and 24 hours. Rh123^high^ fraction at 6 hours was found to be ~42 (p = 0.048), 42 (p = 0.003), 47(p = 0.009) and 38% (p = 0.033) for HPCs, KSL, ST-HSCs and LT-HSCs respectively which further lowered to 22 (p = 0.031), 22 (p = 0.0001), 43 (p = 0.004) and 24%; (p = 0.0004) after 24 hours. However, HMS ameliorated TBI induced changes in MMP and a higher number of KSL and ST-HSCs with Rh123^high^ status were observed both at 6 hours (55; p = 0.025 and 68%; p = 0.022) and 24 h (59; p = 0.0004 and 61%; p = 0.018). In case of HPCs, HMS influenced the MMP status in a complex manner and at 6 hours 79% of progenitors were found to with high MMP which quickly declined to 24% after 24 hours. Interestingly, HMS did not prevent radiation induced loss of MMP in LT-HSCs at 6 hours (39% vs 38% in case of TBI group) but after 24 hours more number of LT-HSCs (44%) with high MMP were present as compared to TBI animals (24%). Administration of AF further enhanced the protective effect of HMS and rescued KSL (54%), ST-HSCs (63%) and LT-HSCs (39%) from radiation induced loss of MMP. Similar to HMS alone, HMS + AF did not prevent radiation induced loss of MMP in HPCs. As radiation induced genomic damage and instability compromises both the short and long term repopulation ability of different subclasses of HSPCs^45^, the DNA damage in the surviving HSPCs was evaluated. In view of recent evidences suggesting that the observed gamma H2AX foci, a marker for assessing DNA damage, in HSCs may mark cellular events distinct from DNA damage^46,47^ we sought to quantify the DNA damage directly in Lin^−^ bone marrow mononuclear cells (BMMNCs) using alkaline comet assay. TBI significantly increased Olive Tail moment (OTM), a reliable end point for DNA damage, when compared to background damage in control (Fig. 5a) after 6 hours which persisted even after 24 hours. However, after 24 hours apparently there were more number of cells with higher OTM when compared to 6 hour time point. HMS significantly ameliorated radiation induced DNA damage in Lin^−^ BMMNCs after 6 hours which persisted even after 24 hours. AF treatment further enhanced the ameliorative effect of HMS and after 24 hours the damage levels were found to be similar to that of control animals (Fig. 5a). Corroborating with DNA damage results, TBI significantly increased the number of annexin V^+^ cells in all four subsets of HSPCs analysed 24 hours later in vehicle treated animals (Fig. 5b–e, Supplementary Fig. 10c). Within different subsets of HSPCs, LT-HSCs were found to be comparatively resistant to TBI induced apoptosis. HMS significantly neutralized TBI induced apoptosis in KSL, ST and LT-HSCs subsets of HSPCs but did not rescue the progenitor cells (HPCs). Animals treated with AMP followed by AF also showed a trend similar to TBI animals treated with AMP only (Fig. 5a).

### HMS preserves c-Kit^low^ HSPCs

Low surface c-Kit levels mark deeply quiescent HSCs with long term reconstitution potential while increased c-Kit levels indicate their proliferation status^48,49^. To assess the influence of TBI and HMS, the expression of c-Kit in annexin V^−^ and annexin V^+^ subsets of different HSPCs was monitored (Fig. 6a-d, Supplementary Fig. 11a). During steady state the surface c-Kit levels in annexin V^−^ fraction varied unevenly, over a log fold, with maximal surface levels observed in LT-HSCs when compared to progenitors (p = 0.001) (Supplementary Fig. 11b). TBI significantly enhanced c-Kit expression in different subsets of annexin V^−^ HSPCs (p = 0.03, 0.0001, 0.016, 0.0004 for HPCs, KSL, ST- and LT-HSCs respectively when compared to control group) (Fig. 5). HMS countered this increase in all HSPCs except HPCs (Fig. 6a–d). AF further reinforced the effect of HMS and, except in HPC, the surface c-Kit levels in different annexin V^−^ HSPCs were found to be similar to that of control animals. The changes in c-Kit expression was more complex in annexin V^+^ fractions. During steady state, different subsets of annexin V^+^ fractions HSPCs exhibited an apparent increase in the c-Kit expression when compared with corresponding annexin V^−^ fraction. (p = 0.034, 0.127, 0.185, 0.001 for HPCs, KSL, ST and LT-HSCs respectively). Interestingly, irrespective of treatment annexin V^+^ HSPCs have exhibited c-Kit high status (Fig. 6a–d).

### HMS increases the serum levels of haematopoietic cytokines and modulates the balance between pro and anti-inflammatory cytokines

**Figure 7:**
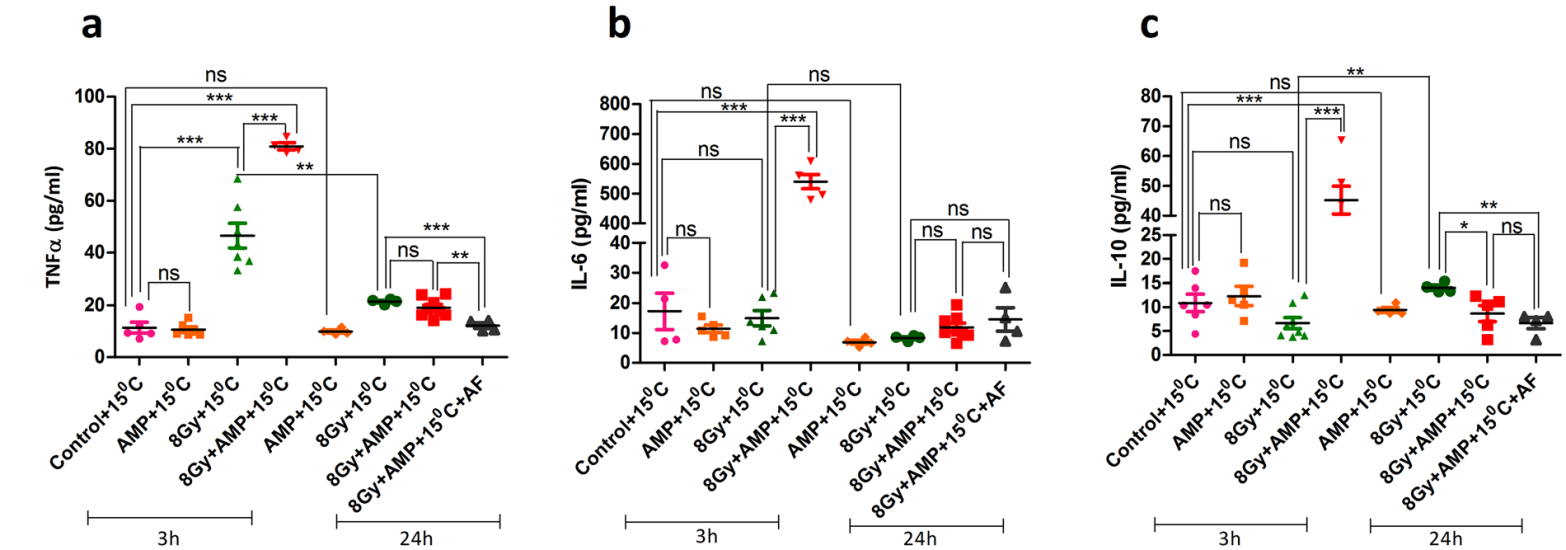
Changes in TNF-alpha, IL-6 and IL-10 levels in serum, collected at different time points after varied treatments. After 3 and 24 hours of different treatment blood was collected, serum was harvested and levels of TNF-α (**a**) IL-6 (**b**) IL-10 (**c**) were quantified flow cytometrically using cytometric bead array. Each value is a mean ± SEM (n = 6 mice/group) and comparisons were made between indicated groups. *p < 0.05, **p < 0.01, ***p < 0.001. AF represents amifostine.

TBI significantly elevated the levels of pro-inflammatory cytokines [TNF-α (~4 fold over control, p = 0.0002), MCP-1 (7.3 fold, p = 0.0001), IL12p70 (3.4 fold, p = 0.0003)] while it did not affect the levels of anti-inflammatory cytokines evaluated in this study [IL-6 (0.86 fold; p = 0.694 when compared to control) and IL-10 (~0.6 fold; p = 0.068)] after 3 hours (Fig. 7a–c, Supplementary Fig. 12). The levels of all cytokines reduced to that of control animals 24 hours after TBI, except MCP-1, which was higher than control group (p = 0.0001) (Supplementary Fig. 12a). HMS significantly reduced TBI induced increase in the levels of circulating IL12p70 (~0.5 folds; p = 0.001 when compared to TBI animals). However, HMS did not affect TBI induced changes in the levels of MCP-1 [~10 fold vs control (p = 0.0001); ~1.3 fold vs TBI group; p = 0.001] and TNF-α [~7 fold vs control (p = 0.0001); ~1.7 folds TBI group; p = 0.241] respectively in TBI animals in 3 hours. Interestingly, HMS countered TBI effect and elevated the levels of IL-6 (~31 and ~36 folds more than control and TBI groups respectively) and IL-10 (~4 and 6.8 folds more than control and TBI groups respectively). After 24 hours the levels of all the cytokines were similar to that of control animals. AF reinforced the effect of HMS and, after 24 hours, further reduced the levels of TNF-α (p = 0.0002; when compared with TBI animals). None of the treatments tried in this study changed the IFN-γ levels both after 3 and 24 hours (data not shown). TNF-α is known to stimulate the expression of IL-6 which in turn replaces TNF-α, a highly toxic cytokine^50,51^ and their ratio (IL-6/TNF-α) is indicative of prevailing inflammatory status. TBI elevated the pro-inflammatory status and the IL-6/TNF-α ratio was found to be significantly lower than the control group after 3 hours (p = 0.0093) (Supplementary Fig. 12c). As deduced from the assessment of individual cytokines, HMS ameliorated TBI induced pro-inflammatory status and the IL-6/TNF-α ratio was found to be significantly higher than both TBI (p = 0.0001) and control (p = 0.02) groups. Even after 24 hours, TBI + HMS group has shown a persisting anti-inflammatory environment when compared to TBI animals treated with vehicle which showed a persisting pro-inflammatory status. However, when compared to control cohort, HMS did not completely normalize the inflammatory status after 24 hours. Also, AF did not further affect the ratio any further to the anti-inflammatory status observed in case of HMS group (p = 0.06).

### Clinically relevant mild HMS also effectively mitigates TBI induced lethality in mice

**Figure 8:**
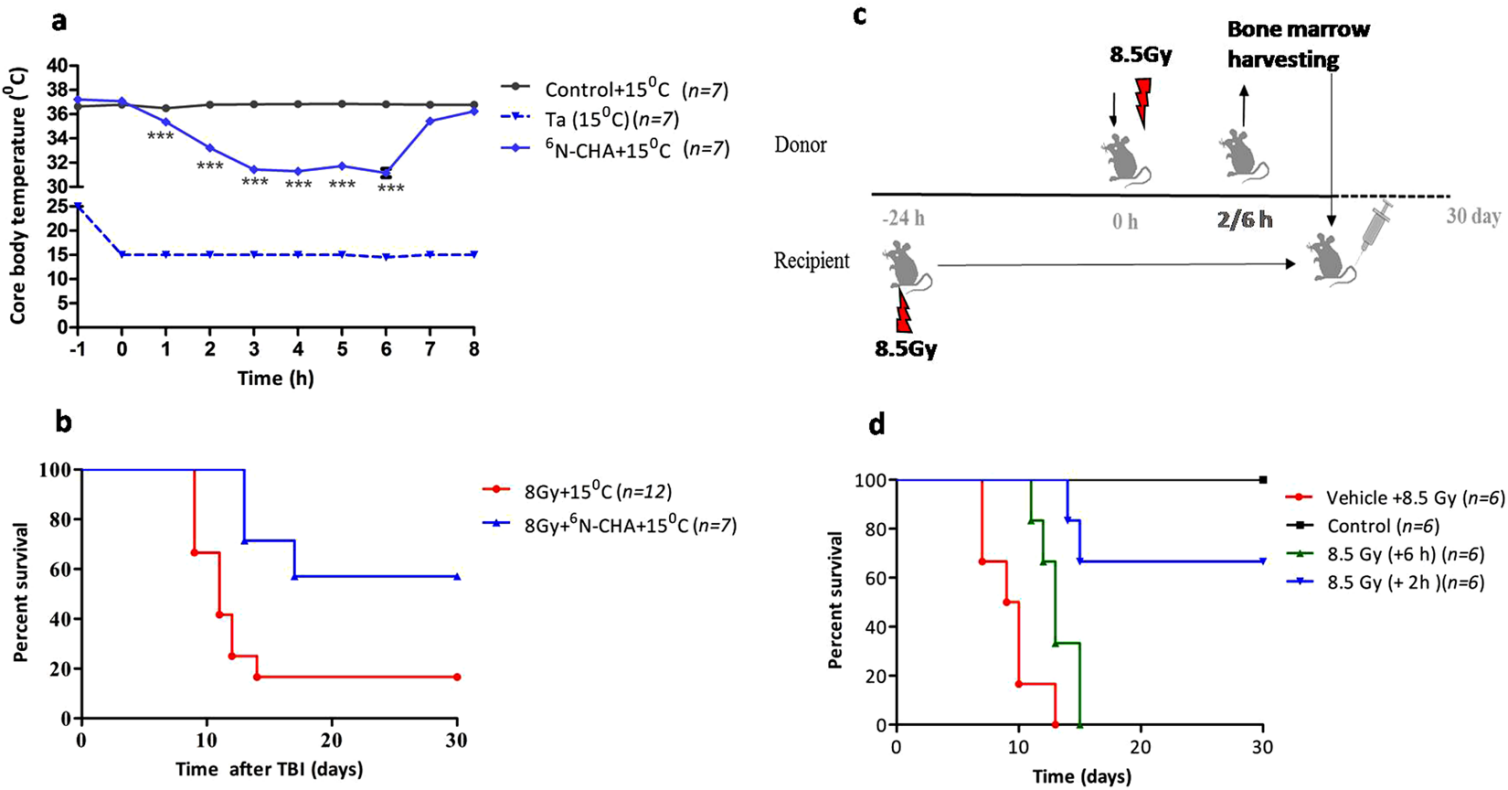
A1AR agonist induced HMS mitigates lethal effects in mice against TBI. B6 male mice were exposed to 8Gy of ^60^Co-γ rays and 60 minutes later either vehicle or CHA (1 mg/kg b.w.) or SJNP-1 (100 mg/kg b.w.) were administered through intraperitoneal or oral route followed by placing them at 15 °C for 6 hours. (**a**) Changes in T_c_ of mice over a period of 8 hours (two way ANOVA after Boneferoni post-test: F = 49.78 and P < 0.0001 for interaction, F = 122.00 and P < 0.0001 for time, F = 3.632 and P < 0.0001 for treatment, ***p < 0.0001 and ^#^p > 0.05 when compared to control + 15 °C). (**b**) Kaplan-Meier plots of 30 days survival of mice. CAF represents caffeine. Bone marrow collected shortly after TBI retains radiomitigative potential. (**c**) Schema of experimental set up for bone marrow collection and syngeneic bone marrow transplantation. Bone marrow was collected 2 or 6 hours after TBI (8.5 Gy), and infused (1 × 10^6^ total nucleated cells) into recipient mice which were lethally irradiated 24 hours before. (**d**) Kaplan-Meier plot showing survival rates of recipient mice transplanted with syngeneic bone marrow collected 2 or 6 hours after lethal irradiation.

Mild HMS with T_c_ reduced to ~32 °C is in clinical practice for the management of cardiac arrest patients^52^. As reduction of T_c_ below 30 °C in patients is associated with increased risk, the radiomitigative effect of clinically relevant reduction in T_c_ (30–32 °C) was tested using CHA, which was previously reported to induce clinically relevant hypothermic state in rodents^53^ (Fig. 8a,b). Corroborating with the reported results, administration of a single dose of CHA and then keeping the animals at T_a_ of 15 °C reduced T_c_ gradually and nadir (~32 °C) was reached after 2 hours which persisted for the duration of the experiment (6 hours) (Fig. 8a). As was observed with AMP, about 2 hours after shifting of animals to room temperature the T_c_ was recovered to euthermic levels. After establishing the mild hypothermia inducing ability, the radiomitigative effect of CHA was evaluated (Fig. 8b). CHA (1 mg/kg b.w.) administered 1 hour after TBI rescued mice from radiation induce lethality (~35% survival advantage over TBI control).

### Bone marrow from lethally irradiated mice collected prior to ensuing inflammatory wave retains radiomitgative potential

The results from this study suggests that pro-inflammatory environment peaks 3 hours after TBI and significantly subsides by 24 hours. HMS exerted the mitigative action by preserving the bone marrow HSPCs from TBI induced inflammatory milieu. To test this in fact is the case and also due to obvious implications in autologous BMT, bone marrow aspirated soon after TBI (2 or 6 hours) was tested for its ability to rescue recipients from lethal TBI (8.5 Gy) (Fig. 8c-d). All animals which received TBI followed by vehicle at 24 hours died within 12 days (Fig. 8d). Transplantation of total bone marrow (1 × 10^6^ cell/ animal), 24 hours after TBI, collected from untreated animals effectively protected all the animals from radiation induced lethality. Transplantation of bone marrow grafts (1 × 10^6^ cell/ animal) harvested 2 hours after TBI (8.5 Gy) resulted in 65% of animals surviving the duration of the experiment (30 days). While animals which received bone marrow grafts harvested 6 hours after TBI did not show any significant improvement in survival when compared to vehicle treated irradiated groups.

## Discussion

The results demonstrate that irradiated animals respond to AMP triggered HMS akin to normal counterparts which is critical for its utility in translating to radiation exigencies. Previously, Daneils *et al*. (2010) have shown using genetically modified mouse models that the modulation of purine nucleotide pool in erythrocytes and increased production of 2,3 bisphospho glycerate and allosteric modulation of oxygen binding to haemoglobin plays an important role in induction of hypometabolic state^37^. However, central activation of adenosine receptor following AMP administration has also been proposed by other investigators^16,39,40^. Consistent with earlier reports, intraperitoneal pre-administration of caffeine abrogated the effect of AMP, suggesting possible involvement of central activation of adenosine receptor in AMP induced HMS (Fig. 1d). However, It is interesting to note that i.c.v administration (500 µg/animal) resulted in significantly lesser reduction in T_c_ when compared to i.p. administration (0.5 mg/g bw). This difference could be attributed to possible involvement of impaired oxygen delivery due to changes in glycolysis in erythrocytes apart from central activation of adenosine receptors in case of i.p. administration which might have resulted in deeper reduction in T_c_. The dose used for i.c.v. administration was more than what was used in earlier reports^40^, suggesting that AMP dose may not be a factor contributing to reduced T_c_ observed in case of animal which received AMP into i.c.v. region.

The disappearance of different subsets of WBCs from peripheral blood during HMS is akin to a situation of immune compromised state^54^ which raises concerns about its utility in managing radiation over exposure victims. In fact, infection has shown to be a common complication of patients undergoing therapeutic hypothermia^55^. However, lack of any disease symptoms in mice which underwent HMS suggests that transient disappearance and resting immune status during a 6 hours HMS may not have adverse effects, suggesting that the approach may have practical utility. It could be attributed to the shorter duration for which the animals were in HMS unlike hibernators or during therapeutic hypothermia where the duration is much longer.

AMP is known to protect mice when administered 15–60 min prior to lethal TBI and induction of hypoxia and enhanced post-irradiation repair have been suggested to be the responsible mechanisms^56–58^. Hypoxic milieu restricts the quality and quantity of free radicals generated upon irradiation and has been suggested to be the mechanism of action for a number of radioprotectors including AMP^34,56^. The present study suggests that apart from the persistent HMS, the reduction in generation of secondary free radicals due to the induction of hypoxia seems to be contributing to the overall radiomitigative effect. Elevated expression of HIF-1α, a reliable marker of hypoxia, both in brain (in frontal cortex) and in femur (bone marrow) supports the notion. In fact it is well known that HMS induced during hibernation or after induction of synthetic torpor leads to induction of tissue hypoxia. The inability of the transient reduction in T_c_, in AMP treated animals placed at 25 °C, to mitigate radiation induced lethality supports the notion that persistent HMS is critical but nevertheless HMS mediated tissue hypoxia seems to be a significant contributory factor.

The ability of moderate hypothermia, with T_c_ reducing to ~32 °C, to mitigate radiation induced lethality suggests that persistent HMS is a critical determinant rather than the depth. This is in line with the beneficial effect of clinically practiced therapeutic hypothermia^52^. Apart from the duration, the timing of induction also seems to be important as induction of HMS later than 3 hours after TBI did not mitigate radiation induced lethality (data not shown). This suggests that initial 3–4 hours are critical for the success of HMS as radiomitigative strategy and is in line with earlier observations that the timing determines success of a given mitigative strategy^59^. Antioxidants are effective mostly when administered prior to irradiation, repair enhancing agents effective when administered shortly after irradiation and cell proliferation inducing agents are effective when administered from 24 hours to few days after irradiation^25,59^. The results also suggests that HMS modulated early events which contribute to the loss of HSCs. Among different deleterious events contributing to the loss of HSCs during early post-irradiation scenario, the elevated levels of inflammogens, loco regional inflammation and protracted oxidative stress seems to be the key players^60–62^. We supported this by showing that HMS shifts the pro- and ant-inflammatory balance towards later by increasing the expression of IL-6 and IL-10 leading to the rescue of different HSPCs in bone marrow. This is in line with earlier reports that systemic hypothermia elevates IL-6 levels^63–65^. However, enhanced proliferation of surviving stem cells contributing to the increased number of HSPCs observed after 24 hours in TBI + HMS group cannot be ruled out. As AMP mediated AR signalling, IL-6 and IL-10 are known to regulate and expand HSPCs^66,67^.

HSCs, post irradiation, are known to exit dormancy and enter into proliferation phase^68^. The better hematopoietic recovery observed in animals which have received AF 8 hours after induction of HMS could be attributed to the free radical scavenging and cytoprotective effects of AF^69^ which probably effectively countered the protracted oxidative stress. CD117 (c-Kit) which marks different HSPCs has also been implicated in regulation of haematopoiesis^70,71^. The inability of lethally irradiated mice to recover from the depressed hematopoietic system could be attributed to the significant increase in the expression of c-Kit. This assumption is in line with earlier studies, with gain and loss-of-function c-Kit mutants, suggesting that subtle changes in c-Kit signalling profoundly impacts HSCs function^72,73^. More recently, Shin *et al*. have shown that c-Kit^high^ HSCs selectively undergo apoptosis in response to 5-FU exposure^34^. The present study also demonstrated that annexin V^+^ cells in different subsets of HSPCs are highly c-Kit positive. Interestingly, the SEM for MFI of annexin V^+^ fraction was found to be much higher than for corresponding HSPCs in annexin V^−^ fraction indicating that annexin V^−^ cells are more homogeneous in terms of c-Kit expression when compared to apoptotic fraction. It is interesting to evaluate whether cells at different stages of the apoptotic cascade exhibits differences in the expression of surface c-Kit. It seems that HMS delayed the entry of different subsets of HSPCs (except HPCs) into proliferation phase post-irradiation thereby preventing them from being exposed to ensuing inflammogens and protracted oxidative stress resulting in increased survival. This assumption is in line with the observation that HMS prevented radiation induced increased expression of c-Kit and largely retained different subsets of HSPCs in c-Kit low status. Similarly, the loss of different subsets of HSPCs in case of irradiated animals could be attributed to the increased entry into division phase, soon after irradiation, which in fact is known to result in their loss^68^.

In this study AMP was found to be effective when administered, 1–3 hours post-irradiation which indicates that sufficient number of undamaged and functionally competent quiescent HSCs survive even after lethal irradiation which with time succumb to ensuing inflammatory wave. This is in line with a recent report by Ishida *et al*. who reported that elevated TNF-α levels in inflamed bone marrow contribute to compromised reconstitution of donor bone marrow^74^. We supported this by demonstrating that transplantation of bone marrow, collected soon after lethal TBI but prior to initiation of radiation induced inflammatory wave (within 3 hours), can rescue lethally irradiated mice. The inability of the bone marrow collected 6 hours after lethal irradiation to protect mice from radiation could be attributed to irreversible loss due to ensuing inflammatory milieu in bone marrow. However, mobilization of surviving HSPCs to extra-medullary hematopoietic sites^75^ leading to reduced number of critical HSPCs in bone marrow collected 6 hours after TBI cannot be ruled out. The countermeasures which can mitigate radiation effects when administered at least 24 hours later are expected to be relevant for mass radiation exposures and the requirement for induction of HMS soon after TBI (within 3 hours) is a critical hurdle in the practical utility of the current approach. However, considering the well accepted clinical practice of therapeutic hypothermia, the immediate translational advantage in using AMP or other small molecules seem to be promising which warrants further investigations in higher mammals.

## Electronic supplementary material

Video 1

Dataset-1
