# Supplementary material for: Pharmacologically induced reversible hypometabolic state mitigates radiation induced lethality in mice
Source: Sci Rep. 2017 Nov 2;7:14900. doi: 10.1038/s41598-017-15002-7 (PMC5668348; doi:10.1038/s41598-017-15002-7)
Supplement: Supplementary file 2 — Dataset-1 [file 41598_2017_15002_MOESM2_ESM.doc]

**Supporting Information**

Pharmacologically induced reversible hypometabolic state mitigates radiation induced lethality in mice

Subhajit Ghosh^1&2^, Namita Indracanti^1^, Jayadev Joshi^1&2^, Jharna Ray^2^,

Prem Kumar Indraganti^1*^

^1^Division of Radiation Biosciences,

Institute of Nuclear Medicine and Allied Sciences,

Brig SK Majumdar Road, Timarpur, Delhi-INDIA

^2^S.N.Pradhan Centre for Neuroscience-University of Calcutta, Kolkata-INDIA

*Correspondence and requests for materials should be addressed:

Dr. Prem Kumar Indraganti email: [prem_indra@yahoo.co.in](mailto:prem_indra@yahoo.co.in)

**Methods**

**Induction of HMS in mice**

T_c_ was measured manually on an hourly basis for the entire duration of the study (8 hours) using a rectal probe (RET-3, Braintree Scientific Inc. MA, USA). Changes in T_s_ was monitored using a thermal imaging camera (HTI1200, Wahl Inc. Instruments, NC, USA) mounted over the top of the cage grid and static dorsal thermal images were captured at different time points. Changes in the peripheral blood flow during HMS i.e microcirculation were directly visualised microscopically in the ear of living animals. For better visualization of peripheral microcirculation, albino (BALB/c) strain mice were used and a day before imaging, hair in the ear were removed by applying commercially available depilating cream (Veet, Rickett Benckiser, Mumbai, India) and gently wiping with moist cotton. Caution was taken to prevent excessive pressure and resultant damage to the vasculature^76^. Those animals which inadvertently receive such damage to the vasculature were removed from the study. The euthermic group of animals were anaesthetised [(7 units of ketamine (80 mg/kg b.w.) and xylazine (10 mg/kg b.w.)] prior to imaging and videography of the ear. The animals (anaesthetised control or similarly treated animals in the HMS) were placed on the microscope stage in such a way that their ear lied on a glass slide (76×25 mm, Blue Star, Mumbai, India) with head positioned on to one side. Another slide was placed over the ear without exerting any pressure and positioned on the microscope stage and the ear was then viewed by trans-illumination from below (SZX16, Olympus, USA) at 1.5× magnification.

The physical activity of the mice during the course of HMS was measured by video recording at different time points (2 minutes each)^77^ using a camera adjusted at a fixed height of 60 cm from the base of the cage. The recorded video was processed using a computer vison based application, developed in house using Python based algorithm. The script was written to assess the video frame by frame and sequential frames were subtracted to generate a difference matrix for two subsequent frames. Finally the mean values, which represents signal movement, within the difference matrix were calculated and the activity was presented as movement index (MI).

Induction of HMS, at biochemical level, was assessed by monitoring the changes in the levels of total tissue (liver) adenosine 5′-triphosphate at different time intervals (0, 1, 3, 6 or 8 hours) after administration of AMP using an ATP bioluminescent assay kit (FLAA-1KT: Sigma, MO, USA). For measuring blood glucose (BG) levels during HMS, blood was collected 6 hours after administration of AMP (T_a_ 15 or 25 ^0^C) through cardiac puncture and BG was measured using commercially available glucometer (Accu Chek, Roche, Mannheim, Germany) essentially following the manufacturers recommendations. For assessing the impact of caffeine on AMP mediated HMS, 40 mg/kg b.w. of caffeine was injected 10 minutes prior to AMP administration. To avoid possible impact of mode of blood collection on BG levels, blood was consistently collected from heart puncture^78^. Pyruvate and L-lactate in plasma was measured using pyruvate and L-lactate assay kits respectively (abcam, Cambridge, USA) and the concentration was determined suing a standard curve prepared using L(+)-Lactate and sodium pyruvate respectively. The concentrations of glucose, pyruvate and lactate was expressed as mg/dl of blood, µM/ 5 µl of plasma respectively. For haematological profiles, at different time points after varied treatments, animals were killed and blood was drawn by cardiac puncture and collected into EDTA containing Eppendorf tubes. Haematological profiling was done within 1 hour of collection using an automated haematology analyser (Celltac α, Nihon Kohden, Japan).

**Animals, ICV Injection of AMP and measurement of T_b_**

For establishing the role of central activation of AR in the induction of hypometabolic state by AMP, direct intracerebroventricular (i.c.v) injection of AMP was given to 8-10 weeks old female Sprague Dawley rats weighing 180±10g. i.c.v. microinjection of AMP was accomplished using glass micropipettes attached to a pressure injection system. The rat brain atlas was used for selecting the brain coordinates using stereotactic apparatus (Stoelting Co., Wood Dale, IL, USA), the internal cannula was connected to a gas-tight syringe positioned in the implanted guide cannula. AMP (500 µg/animal in a volume of 5 µl) was injected i.c.v (X= -0.34, Y= +1.2, Z= +3.8 mm from bregma) at a rate of 1µl/minute in anesthetized rat (80 mg/kg b.w. of ketamine and 10 mg/kg b.w. of xylaxine). For assessing the involvement of adenosine receptors, caffeine (45 mg/kg b.w.) was administered through intraperitoneal route 10 minutes prior to i.c.v. injection of AMP. After i.c.v. injections of AMP alone or caffeine +AMP, the animals were shifted to either a T_a_ of 15°C or 25°C. T_b_ was measured initially 30 minutes after i.c.v. injections and thereafter measurements were done on an hourly basis. The surgical procedures and post-operative care was done essentially following the method reported by DeVos and Miller (2015)^79^.

**Spatial dynamics of lymphocytes during HMS-*In vivo* optical imaging**

Spatial dynamics of lymphocytes during the course of HMS was monitored flow cytometrically or by *in vivo* optical imaging of carboxyﬂuorescein diacetate succinimidyl ester (CFSE) labelled lymphocytes. CFSE is an amine reactive cell permeable fluorescent probe used widely to label cells for applications including lumphocyte division, cell tracking and hematopoietic stem cell homing. Total lymphocyte fraction was harvested by centrifuging EDTA treated blood, drawn from cardiac puncture, on Ficoll gradient. Lymphocytes (1×10^6^/ml) were labelled with CFSE (5 μM) in PBS+5% FBS for 15 minutes at 37°C in dark with intermittent mixing. Labelling was terminated by adding 5 ml of ice cold PBS+5% FBS and washed thoroughly to remove unbound CFSE from the sample^80^. The labelled lymphocytes (0.5×10^6^ cells/animal) were injected through tail vein and 30 minutes later either vehicle or AMP was administered and animals were kept at 15°C. In a different set of animals, caffeine, a pan AR antagonist, was administered 30 minutes prior to infusion of labelled lymphocytes and induction of HMS. 2 hours after AMP injection, animals were killed and blood was drawn through cardiac puncture and collected into EDTA containing tubes. RBC was lysed using 1×ACK (ammonium chloride potassium) solution and samples were acquired using a flow cytometer (BD Accuri C6, San Jose, CA). The granulocyte and lymphocyte populations were determined using a back gating approach where a control sample was stained for granulocytes (Gr1-PerCP/Cy5.5; 0.5 µl/10^6^ cells; Biolegend, CA, USA) and lymphocytes (CD45-APC; 0.5µl/10^6^ cells, Biolegend). For *in vivo* imaging, 2 hours after AMP administration, animals were positioned ventrally under light anaesthesia (ketamine+xylazine), and CFSE labelled cells were imaged (ex/em: 488/525 nm) using an *in vivo* optical imaging platform (Kodak Imaging system FX, CA, USA). The conditions for acquiring the images are, exposure time: 10 seconds, lamp intensity: high and with no binning.

**Cell counts**

Bone marrow samples harvested from mice after different treatments were stained and acquired within 30 minutes of collection using a flow cytometer (BD Accuri® C6)^81^. For accurate volumetric measurements, fluid calibration was done on the day of counting, following the manufacturer's recommendations. The absolute volumetric count was calculated as follows: Total cell count (cells/µL) = [no. of viable cells of interest/ measured sample volume] × dilution factor.

**Cytologic evaluation of bone marrow**

To determine the effect of AMP induced HMS on radiation induced damage to hematopoietic system, the femurs were collected 24 hours after different treatments including non-irradiated control (0 Gy+T_a_ 15^0^C), irradiation control (8Gy+T_a_ 15^0^C), irradiated and AMP treated (8Gy+AMP+T_a_ 15^0^C) and then subsequently bone marrow smears were made using a paint brush. Bone marrow cells were stained using May-Grünwald Giemsa stain (MGS) (MGS is a Romanowsky stain comprising of May-Grünwald and Giemsa. This pan-optic staining gives brilliant staining to the nuclei, cytoplasmic granules and other bodies and is widely used for staining of blood and bone marrow smears) and a minimum of 200 nucleated cells per animal were counted from each treatment group. The radiation-induced damage was evaluated as changes in myeloid and erythroid cell numbers^82^.

**Enumeration of bone marrow HSPCs**

The collected bone marrow was treated with 1× ice cold RBC lysis solution (ACK: 155 mM ammonium chloride, 1 mM potassium bicarbonate, 0.1 mM EDTA) for 2 minutes at 4°C to remove RBCs. For lineage depletion (Lin^-^), bone marrow mononuclear cells (BMMNCs) were incubated with biotin conjugated antibodies against murine CD5, Mac-1, CD45R/B220, Ter-119, and Gr-1 (Lineage Cell Depletion Kit mouse, Miltenyi Biotec GmbH, Germany). Mature myeloid and lymphoid cells were depleted by incubating them with anti-biotin magnetic micro-beads and were finally separated using MS columns and MACS separator (Miltenyi Biotec GmbH, Germany) following manufacturer’s recommendations. The Lin^-^ cell fraction was washed in PBS (+2% FBS) and the cell number was counted volumetrically as described earlier. For analysis of different HSPCs, Lin^-^fraction was pre-incubated with anti-CD16/32 antibody (0.5 µl/10^6^ cells; Biolegend, CA, USA) to block the Fcγ receptors and was then stained with anti-Sca1-PE (1 µl/10^6^ cells; Biolegend), c-Kit-APC-Cy7 (0.5 µl/10^6^ cells, Biolegend) and CD34-PerCP/Cy5.5 (0.8 µl/10^6^ cells; Biolegend) and this procedure was carried out on ice in the dark. The frequencies of hematopoietic progenitor cells (HPCs) (Lin^-^ Sca1^+^c-kit^+^ cells), KSL (Lin^-^ Sca1^+^c-Kit^+^ cells), short term-hematopoietic stem cells (ST-HSCs) (Lin^-^Sca1^+^c-Kit^+^ CD34^+^cells)^83^ and long term-hematopoietic stem cells (LT-HSCs) (Lin^-^Sca1^+^c-Kit^+^CD34^-^cells)84 were analysed using BD Accuri C6 software. Appropriate isotype and single positive controls were also acquired whenever required for compensation and at least 20,000 cells were acquired for each sample. For the analysis of changes in the mitochondrial membrane potential in different HSPCs, after treating with different antibodies for 45 minutes, rhodamine 123 (10 nM; Sigma, St Louis, MO) was added and the samples were immediately acquired. Similarly, after treating the samples with antibodies for different surface markers, cells were resuspended in binding buffer containing anti annexin V-FITC (3 µl/10^6^ cells; Sigma, St Louis, MO) and further incubated for 15 minutes in the dark on ice. Thereafter cells were then washed and acquired. The numbers of different HSPCs populations were calculated and presented as frequencies per million BMMNCs.

**Alkaline comet assay in Lin^-^ BMMNCs**

Alkaline comet assay, for detecting frank as well as alkali labile DNA damage, was performed in Lin^-^ BM-MNC as previously described^85^. Briefly, 5000 Lin^-^BMMNCs were mixed in 150 µl of 0.5% low melting point agarose (Sigma-, St Louis, MO) in PBS and layered onto 1% normal melting point agarose (1%) pre-coated slides. Cells were lysed over night at 4°C in alkaline lysis solution (2.5 M NaCl, 100 mM Na_2_EDTA, 0.01 M Tris, 10% DMSO, 1% triton-x-100, pH 10.0). The slides were washed and incubated in freshly prepared alkaline unwinding solution (300 mM NaOH, 1 mM EDTA, pH 13.0) for 20 minutes and were run in the same conditions at 0.6 V/cm for 25 min at 4°C. Slides were neutralized in tris buffered solution (0.4 M tris, pH 7.5) and stained in propidium iodide (a fluorescent DNA intercalator) solution (25 µg/ml) for 20 minutes. Images of individual nuclei were taken using a fluorescence microscope (Zoe, Biorad, Hercules, CA) and the data was analysed using the CASP software (http://casplab.com/). At least 100 nuclei were analysed from each treatment group and Olive tail moment was calculated for expressing the levels of DNA damage^86^.

**Measurements of inflammatory markers in serum**

Blood was collected at 3 and 24 h after different treatments in the Eppendorf tubes without any anticoagulant. Serum was collected by letting the blood stand for 30 minutes at room temperature followed by centrifugation (3000 rpm for 15 minutes at 4°C). The clear serum was collected and stored at -80°C until further analysis. For measuring the levels of inflammatory cytokines, cytometric bead array method was used (BD Cytometric Bead Array (CBA) Mouse Inflammation kit, BD, USA). The levels of mouse Interleukin-6 (IL-6), Interleukin-10 (IL-10), Monocyte Chemoattractant Protein-1 (MCP-1), Interferon-γ (IFN- γ), Tumor Necrosis Factor (TNF-α), and Interleukin-12p70 (IL-12p70) were quantified flow cytometrically following the manufacturer’s recommendations. The correlation coefficient of the standard curve prepared independently before every run for all the cytokines was consistently ≥ 0.98 while the intra assay CV was <20%. The analytes which showed a value more than the threshold limit of the assay were only considered for further analysis.

**Measurement of HIF1α expression in frontal cortex (brain) and femur (bone marrow)**

For assessing the changes in the expression of Hif -1α in brain, frontal cortex was dissected out from the mouse brain and single cell suspension was prepared using a commercially available kit, (Adult brain dissociation Kit, Miltneyi Biotech, USA) following the manufacturer’s recommendations. The resulting single neuronal preparation was fixed in cold 4% paraformaldehyde (PFA) (20 minutes at 4^0^C). Thereafter, the cell suspension was washed three times using binding buffer (PBS+2% BSA+0.1% Tween-20) to remove the traces of PFA followed by re-suspending the cell pellet in 100 µl of binding buffer. Mouse anti-Hif-1α (abcam, Cambridge USA) was added to the cell suspension and was left overnight at 4^0^C. After washing, FITC tagged secondary antibody was added to the cell suspension in the same buffer and incubated for 2 hours at room temperature. Finally the cell suspension was washed to remove the unbound secondary antibody and then the cells were acquired flow cytometrically. 7-AAD was used as a counterstain. Bone marrow was harvested from the femur and single cell suspension was processed as mentioned above for the neuronal cells except that lineage negative cells were isolated and stained with appropriate surface markers (anti c-kit and sca-1) for identifying KSL cells.

**Syngeneic BMT**:

For assessing the radiomitigative potential of bone marrow cells, they were harvested after TBI and were thus syngenically transplanted. Whole bone marrow was harvested from the female B6 mice, sacrificed 2 or 6 hours after lethal TBI (8.5Gy) and the cells were immediately injected (1×10^6^ nucleated cells) via the tail vein into the recipient mice which were lethally irradiated (8.5Gy) 24 hours prior to transplantation. Survival of the animals was recorded for 30 days.

**Results**

**5-AMP induces reversible HMS in un-irradiated and lethally irradiated mice**

Except oral all other routes of administration tried in this study (intramuscular, intraperitoneal or intravenous) were found to be equally effective (Supplementary Fig. 1a). Similarly, AMP induced HMS in lethally irradiated mice in a strain and sex independent fashion (Supplementary Fig. 1b). As the body surface, which is in direct contact with the environment, responds to changes in T_a_ by a variety of means^87^ it was considered interesting to assess how the body T_s_ responds to AMP and different T_a_ (Supplementary Fig. 2a-b). Within 10 minutes of AMP (+T_a_ 25°C) administration the T_s_ reduced and nadir was reached 45 minutes later which thereafter recovered completely by 3 hours. When compared to changes in T_c_, the nadir for T_s_ reached early (45 minutes for T_s_ vs 60 minutes for T_c_) and the recovery was comparable (180 minutes for both T_a_ and T_s_). Unlike T_c_, mice without any treatment that were placed in the T_a_ of 15°C did show a gradual reduction in T_s_ and the nadir (~30°C) reached after 30 minutes which remained at reduced levels for the entire duration of the experiment (till 6 hours). The T_s_ quickly returned to normal levels when the mice were transferred from T_a_ of 15°C to T_a_ of 25°C. Mice which were treated with AMP (+T_a_ 15^0^C) also showed a steep reduction in T_s_ which reached to 30°C within 10 mins while nadir (16°C) was attained in 20 minutes. When compared to T_c_, in this group the nadir (16°C) for T_s_ reached earlier (20 minutes vs 120 minutes). Both un-irradiated and lethally irradiated mice exhibited similar T_s_ kinetics.

**AMP mediated HMS and vasoconstriction**

In mammals, asympathetic noradrenergic vasoconstriction and reduced skin blood flow is the key to prevent excessive heat dissipation during cold exposure^88,89^. To establish the role of peripheral vasoconstriction and changes in blood flow in the reduction of surface body temperature, change in peripheral vascular remodelling and circulation after different treatments were monitored in vivo. To address this, we used the ear of albino mice (BALB/c) to directly visualize the changes in the blood flow before and after different treatments. A neurovascular bundle containing an artery, vein and nerve forms the main supply to mouse ear where the central artery bifurcates into two main arterioles which supply blood to the ear. For measurement of vascular remodelling the right of the main arteriole was used (Supplementary Fig. 3a-b). Mouse ear imaged without any treatments had a clear vasculature with normal blood flow in central artery, the bifurcated main arterioles as well as the arterioles dispersed throughout the pinnae (Supplementary Fig. 3a). Within 15 minutes after AMP (+T_a_ 15^0^C) administration, the caliber of both the central artery and main arterioles reduced significantly (p=0.0004) when compared to untreated control animals (Supplementary Fig. 3b). Moreover, the imaging done at 30, 45 or 60 minutes after treatment did not show any additional change in the caliber of the vasculature and blood flow. Mice which received TBI 60 minutes prior to AMP (+T_a_ 15°C) treatments also showed similar changes in the peripheral vasculature (data not shown). Untreated mice placed at T_a_ of 15°C did show a significant reduction in the arteriole diameter and overall blood flow when compared to untreated control group (p=0.399). However, the reduction in main arteriole diameter and blood flow was less severe than AMP treated animals placed at T_a_ of 15°C (p=0.0001).

**Effect of AMP mediated HMS on movement Index (MI)**

In this study, to induce HMS, mice treated with AMP were placed in an incubator, for exposing them to low T_a_, which thereby prevented direct visualization of changes in behaviour after different treatments. To generate behavioural profiles after different treatments, a 2D cage activity algorithm was developed in house to assess the subtle changes in motor activity (Supplementary Fig. 4a). The 2D movement inside the cage was analysed for the entire duration of the study (8 hours) as a movement Index (MI) which quantifies the motor activity. Mice which received only vehicle and then placed at a T_a_ of 25°C, exhibited normal cage behaviour with frequent movement inside the cage (Supplementary Fig. 4b) while mice treated with vehicle and placed at a T_a_ of 15°C, showed lesser movements and changed the shape of the MI profile albeit to a small extent when compared to vehicle treated animals placed at a T_a_ of 25°C. Interestingly, mice treated with AMP and placed at a T_a_ of 25°C shifted the overall MI profile towards slower movement with intermittent static phases with no MI. Unlike untreated control, both lethally irradiated as well as un-irradiated mice which received AMP and subsequently placed at a T_a_ of 15°C considerably changed the MI profile towards a static phase with MI close to a value of zero. The movement was found to be ceased soon after AMP administration and the static phase persisted until the animals were shifted from T_a_ of 15°C to a T_a_ of 25°C. About 1 hour after the animals were placed at a T_a_ of 25°C, they resumed movement albeit slowly. Caffeine treatment significantly abolished the AMP (+T_a_ 15°C) induced shift in MI towards the static phase. However, the overall MI was found to be towards the slow movement side. It was observed that AMP (+T_a_ 15°C) induced a shift in MI profile towards the static phase in a strain and sex independent fashion (Supplementary Fig. 4c).

**AMP mediated HMS alters carbohydrate metabolism**

AMP catabolizes to ADP by using up cellular ATP, which contributes to overall HMS^27^. Ionizing radiation is known to influence mitochondrial metabolism and ADP-AMP dynamics^90^. It was interesting to investigate the effect of AMP induced HMS on tissue ATP levels in the untreated and lethally irradiated mice (Supplementary Fig. 5a). Consistent with the changes in T_c_, mice which received AMP (+T_a_ 25°C) showed an apparent reduction in ATP levels in liver when measured 1 hour after treatment (p>0.05). Thereafter, ATP level's gradually increased and normal level was achieved within 3 hours. Both irradiated and un-irradiated mice which received AMP (+T_a_ 15°C) treatments showed a steep decrease in the tissue ATP levels which reached nadir after 6 hours of treatment. Caffeine treatment prior to AMP (+T_a_ 15) treatment did not influence the initial reduction observed after 1 hour but it completely abolished the second phase (post 1 hour) of drop in ATP levels.

Consistent with the reduced generation of cellular ATP, AMP mediated HMS resulted in the suppression of metabolic consumption. It was also seen that AMP (+15^0^C) induced significant increase in the levels of blood glucose when observed 6 hours after administration (p=0.0001; when compared with vehicle control (+15^0^C)) (Supplementary Fig. 5b). Irrespective of the incubation temperature, the animals exhibited similar blood glucose levels (p=0.0627). However, interestingly the animals which received AMP (+25^0^C) exhibited significantly lower blood glucose levels when compared to vehicle control (+25^0^C). Caffeine when administered 30 minutes prior to AMP injection, completely abrogated the effect of AMP (+15^0^C) and blood glucose levels were found to be reduced albeit lesser than the vehicle control (+15^0^C) animals (p=0.0013) (Supplementary Fig. 5b). Consistent with the suppressed metabolism resulting in increased plasma glucose levels, animals which received AMP (+15^0^C) exhibited reduced levels of pyruvate (Supplementary Fig. 5c) and lactate (Supplementary Fig. 5d), the end products of glycolysis, when compared to vehicle control (+15^0^C) group (p=0.0190 and 0.0160 for pyruvate and lactate respectively). As was observed with glucose, pre-treatment of caffeine resulted in complete reversal of AMP mediated effect on both pyruvate and lactate (Supplementary Fig. 5c,d ).

**HMS induces tissue hypoxia**

To establish the induction of tissue hypoxia during HMS, Hif-1α expression was studied in neurons of the frontal cortex and bone marrow of femur. Administration of AMP (+T_a_ 15°C) resulted in a significant increase in the expression of Hif-1α (6 hours after administration) both in the frontal cortex (p=0.0128 ) as well as in bone marrow (p=0.0006) when compared to vehicle treated mice maintained at a T_a_ of 15°C, suggesting prevailing hypoxia (Fig. 1 b,c). Interestingly, mice which received AMP and thenceforth placed at T_a_ of 25°C have also shown a significant increase in Hif-1α expression both in the frontal cortex and femur albeit less than the levels observed in AMP (+T_a_ 15°C). Treatment of mice with caffeine 10 minutes prior to the administration of AMP (+T_a_ 15°C) completely abrogated the induced expression of Hif-1α (Fig. 1 b,c). Caffeine similarly ameliorated the Hif-1α expression in mice treated with AMP and placed at T_a_ of 25°C.

**Effect of AMP mediated HMS on reproductive capacity**

As for translational success, the approach needs to be safe without any short or long-term issues, the effect of AMP induced HMS on the reproductive ability and foetal development in mice was studied. Female mice which received AMP (+T_a_ 15°C) a day before being set for mating or after they are pregnant (1 days after appearance of vaginal plug) were monitored for the number of viable pups and development (Supplementary Fig. 6). AMP induced HMS for the duration tried in this study (6 hours), both prior to and during pregnancy, did not alter the normal reproductive ability and the number of viable pups (39 prior to mating vs 30 during pregnancy; p=0.01), and thus the development was found to be normal without any apparent abnormalities.

**HMS affects circulating peripheral WBCs and platelets similarly in both lethally irradiated and un-irradiated mice.**

AMP induced HMS has previously shown to affect the dynamics of circulating WBCs by activating adenosine 2B receptors and decreasing the sphingosine 1-phosphate levels^91^. As moderate doses of irradiation massively depletes circulating lymphocytes, the impact of HMS on their dynamics was studied. Within 1 hour after administration of AMP (+T_a_ 25°C), the number of circulating WBCs, lymphocytes, granulocytes and platelets reduced significantly (p=0.001 when compared to untreated control) which thereafter increased and 3 hours later, the levels were found to be similar to that of untreated control animals (Supplementary Fig. 7a-d). Among the WBCs, lymphocytes have shown much steeper reduction when compared to granulocytes (p=0.001). Both un-irradiated and lethally irradiated mice treated with AMP (+T_a_ 15°C) exhibited, within 1 hour, a steep decrease in the number of circulating WBCs, lymphocytes and granulocytes which reached to minimum after 3 hours and remained at lower levels till 6 hours after which their number began to increase and reached to the normal levels when the animals were shifted to T_a_ 25°C. The number of circulating platelets showed a significant reduction after 3 hours which remained at lower levels till the animals were at T_a_ of 15°C and their number increased to normal levels within 2 hours after they were shifted to T_a_ of 25°C (Supplementary Fig. 7d). Irradiated mice treated with caffeine, prior to administration of AMP (+T_a_ 15°C), completely abolished the AMP and T_a_ of 15°C induced reduction in circulating WBCs, lymphocytes, granulocytes and platelets. To establish the involvement of AR mediated signalling in the disappearance of WBCs, CFSE labelled PBMC injected into mice were quantified flow cytomterically in peripheral blood 3 hours after different treatments (Supplementary Fig. 8). Consistent with the in vivo WBC dynamics, CFSE labelled PBMC also responded to AMP (+T_a_ 15°C) treatment and showed similar dynamics. Pre-administration of caffeine almost completely abolished the disappearance of WBCs. Interestingly, CFSE labelled PBMC treated with caffeine (100 µg/10^6^ PBMC) ex vivo partially, yet significantly, reduced the disappearance of PBMC from peripheral blood (Supplementary Fig. 8a-b) when compared to in vivo caffeine treatment (p=0.035). Earlier reports have suggested that homing in lymphatic system and between endothelial cells is the major reason for the reduction in the number of circulating WBCs and platelets in response to AMP (+T_a_ 15°C) induced HMS^92^. To track their homing and egress, CFSE labelled PBMC were administered into mice and the homing of PBMC was tracked using non-invasive in vivo optical imaging system (Supplementary Fig. 8c). Untreated mice imaged 3 hours later have shown an increased distribution of CFSE labelled PBMC in upper thoracic and upper abdominal region. Lethally irradiated or un-irradiated mice injected with AMP (+T_a_ 15°C) have shown an increased localization of PBMC in cervical, brachial and inguinal lymph nodes. Ex-vivo imaging of the harvested lymph nodes also established the localization of CFSE^+^ PBMC into lymph nodes.

**HMS effectively mitigated TBI induced loss of body weight in mice**

TBI induced significant loss of body weight starting from day 3 (7%) in vehicle treated animals which began to increase gradually and maximal loss (16%) was observed on day 9 (Supplementary Fig. 9). The surviving mice showed a slight improvement and the percent loss reduced to 12% on day 11 which persisted for the entire length of the experiment (day 30). Lethally irradiated mice placed in T_a_ of 15°C showed a gradual loss of body weight which peaked (28%) on day 17. Thereafter the surviving mice showed an increase in body weight and the loss was reduced to 19% persisting for the rest of the experimental duration. However, AMP (+T_a_ 15°C) induced HMS mitigated radiation induced loss of body weight and the animals lost around 7% of their body weight which remained same till 23rd day. Thereafter, the animals have shown recovery and the body weight gradually increased and on day 30 the loss of body weight was found to be 3%. Lethally irradiated mice which received amifostine 8 hours after AMP administration (+T_a_ 15°C) lost body weight gradually from day 1 which peaked on day 5 (18.9%). Thereafter the surviving animals showed recovery and their body weight gradually increased and on day 30 the loss of body weight was 3% of their initial weight.

**HMS improves recovery from radiation induced lympho, granulo, erythrocytopenia and restores spleen cellularity.**

TBI rapidly (within 24 hours) depleted the number of circulating lymphocytes and granulocytes while pharmacologically induced HMS significantly enhanced their recovery from radiation induced depletion Amifostine when administered 8 hours after AMP treatment, apparently further accelerated the recovery observed in TBI+HMS group (Supplementary Fig. 9b,c). TBI significantly reduced the RBC count (7.23×10^6^/µl) on day 30 when compared to control cohort (10×10^6^/µl; p=0.0001) (Supplementary Fig. 9d). However, pharmacologically induced HMS with (9.1×10^6^/µl) or without (8.7×10^6^/µl) AF significantly improved the recovery and the RBC count on day 30 when compared to TBI group (p=0.0001 and 0.0001). The immature lymphocytes in extra-hematopoietic organ like spleen readily underwent interphase cell death, in response to TBI, leading to reduced spleen mass and along with it reduced size. TBI significantly depleted the cellular mass and also reduced the size and mass of the spleen on day 30 (0.067 g) when compared to control animals (0.121g; p=0.009). TBI+HMS group with (0.113 g; p=0.514) or without AF (0.129 g; p=0.792) significantly improved the recovery and the spleen mass was similar to that of control animals on day 30 (Supplementary Fig. 9e).

**References**

76. Billaud, M. et al. A new method for in vivo visualization of vessel remodeling using a near-infrared dye. *Microcirculation* **18**, 163-171 (2011).

77. Kokel, D. et al. Rapid behavior-based identification of neuroactive small molecules in the zebrafish. *Nat. Chem. Biol.* **6**, 231-237 (2010).

78. Togashi, Y. et al. Evaluation of the appropriateness of using glucometers for measuring the blood glucose levels in mice. *Sci Rep*. **6**, 25465 (2016).

79. DeVos, S.L & Miller, T.M. Direct intraventricular delivery of drugs to the rodent central nervous system. *J Vis Exp*. **12(75)** (2013).

80. Quah, B. J., Warren, H. S & Parish, C. R. Monitoring lymphocyte proliferation in vitro and in vivo with the intracellular fluorescent dye carboxyfluorescein diacetate succinimidyl ester. *Nat. Protoc.* **2**, 2049-2056 (2007).

81. Mariani, M. et al. Evaluation of an easy and affordable flow cytometer for volumetric haematopoietic stem cell counting. *Blood Transfus.* **12**, 416-420 (2014).

82. Bolliger AP. Cytologic evaluation of bone marrow in rats: indications, methods, and normal morphology. *Vet. Clin. Pathol*. **33(2),** 58-67 (2004).

83. Osawa, M. et al*.* Long-term lymphohematopoietic reconstitution by a single CD34-low/negative hematopoietic stem cell. *Science* **273**, 242-245 (1996).

84. Ishida, T. et al. Pre-Transplantation Blockade of TNF-alpha-Mediated Oxygen Species Accumulation Protects Hematopoietic Stem Cells. *Stem Cells* **35**, 989-1002 (2017).

85. Olive, P. L & Banath, J. P. The comet assay: a method to measure DNA damage in individual cells. *Nat. Protoc*. **1**, 23-29 (2006).

86. Konca, K. et al. A cross-platform public domain PC image-analysis program for the comet assay. *Mutat. Res.* **34**, 15-20 (2003).

87. Terrien, J., Perret, M., Aujard, F. Behavioral thermoregulation in mammals: a review. *Front. Biosci.* **16**, 1428-1444 (2011).

88. Johnson, J. M., Minson, C. T & Kellogg, D. L. Jr. Cutaneous vasodilator and vasoconstrictor mechanisms in temperature regulation. *Compr. Physiol*. **4,** 33-89 (2014).

89. Charkoudian, N. Mechanisms and modifiers of reflex induced cutaneous vasodilation and vasoconstriction in humans. *J. Appl. Physiol.* **109,** 1221-1228 (2010).

90. Wang, Y. J. et al. Irradiation induced injury reduces energy metabolism in small intestine of Tibet minipigs. *PloS One* **8,** e58970 (2013).

91. Bouma, H. R. et al. Reduction of body temperature governs neutrophil retention in hibernating and non-hibernating animals by margination. *J. Leukoc. Biol*. **94,** 431-437 (2013).

92. Bouma, H. R, et al. 5'-AMP impacts lymphocyte recirculation through activation of A2B receptors. *J. Leukoc. Biol.* **94,** 89-98 (2013).


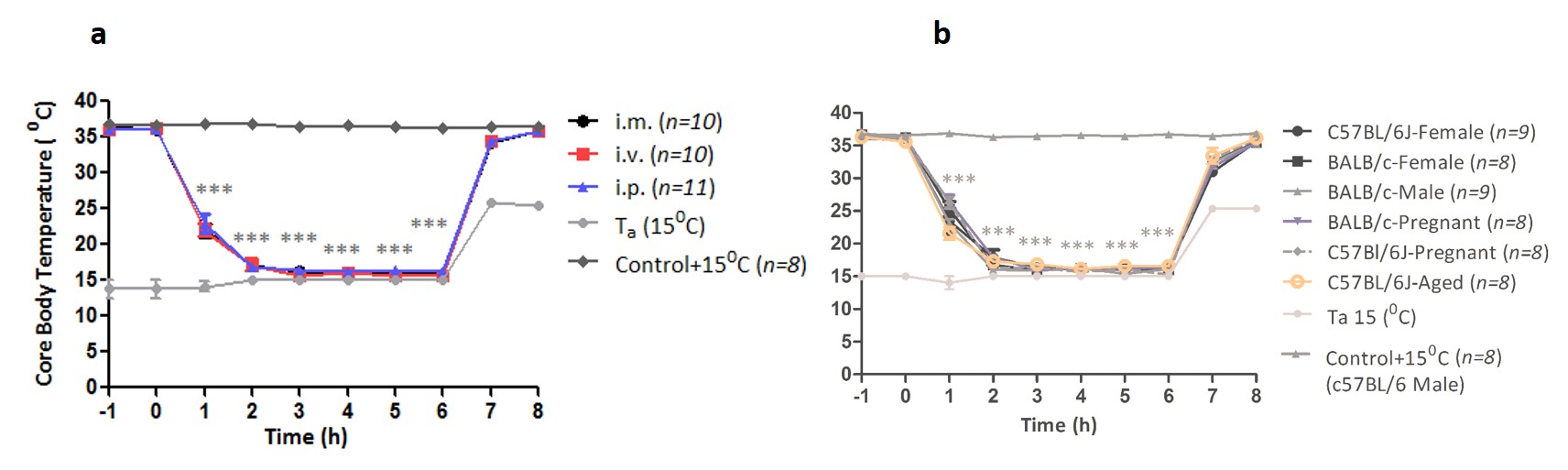


**Supplementary Figure 1: Effect of AMP induced HMS on T_c_.** (**a**) Effect of route of administration on AMP induced HMS. AMP in a total volume of 0.2 ml (for IM route a volume of 0.05 ml) was administered through different routes and immediately placed at a T_a_ of 15°C and changes in T_c_ were monitored (two way ANOVA after Boneferoni post-test: F=117.2and P<0.0001 for interaction, F=1204.0 and P<0.0001 for time, F=473.2 and P<0.0001 for treatment, ***p<0.001 for all AMP treated group at 15^0^C (1-6h) when compared to control+15^0^C). (**b**) Effect of strain, age, sex on AMP induced changes in T_c_. (two way ANOVA after Boneferoni post-test: F=49.61and P<0.0001 for interaction, F=1877.0 and P<0.0001 for time, F=297.5 and P<0.0001 for treatment, ***p<0.001 for all AMP treated group at 15^0^C (1-6 h) when compared to control+15^0^C). Each value represents a mean ± SEM. Caf represents caffeine.


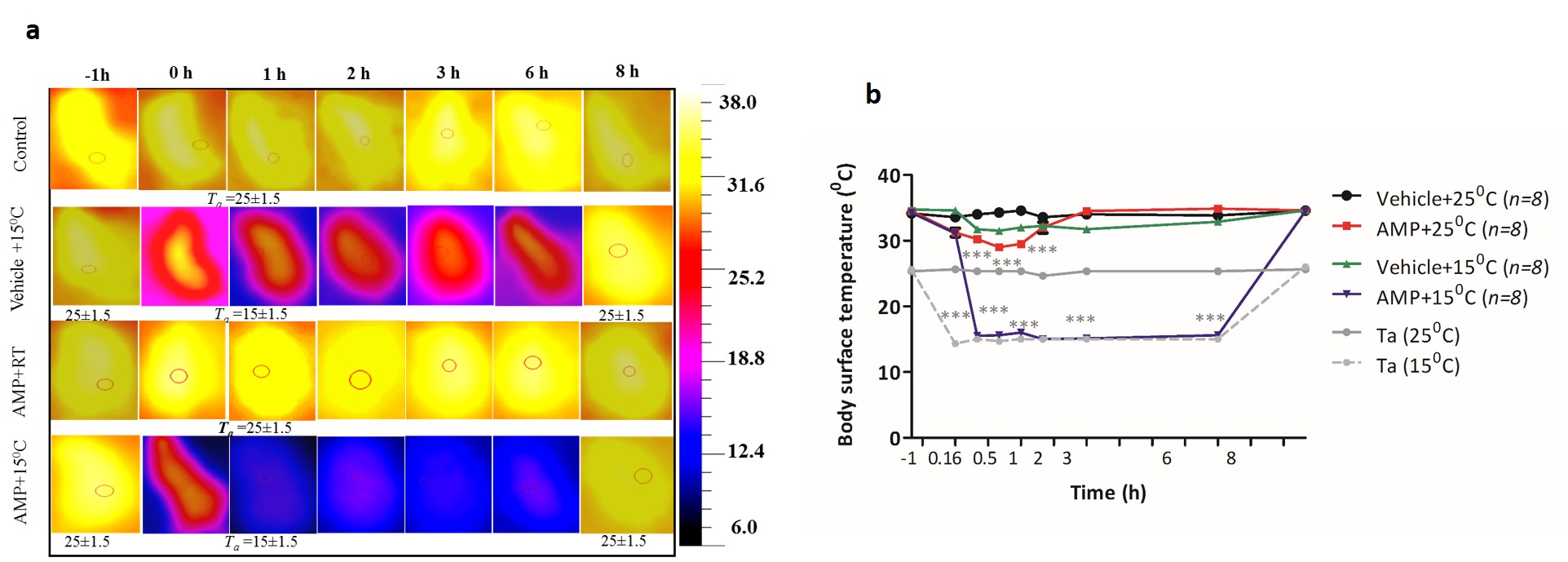


**Supplementary Figure 2: AMP induced HMS and changes in surface body temperature.** B6 male mice were administered with AMP and infrared images were captured at indicated time intervals. (**a**) Representative IR images captured at different time intervals after the indicated treatments. (**b**) Changes in surface body temperature with indicated treatment recorded over a period of 8 hours after administration of AMP (two way ANOVA after Boneferoni post-test: F=197.8and P<0.0001 for interaction, F=353.5 and P<0.0001 for time, F=1704.0 and P<0.0001 for treatment, ***p<0.001 for AMP+15^0^C and AMP+25^0^ when compared to control+15^0^C and control+25^0^C). Each value represents a mean ± SEM.

**
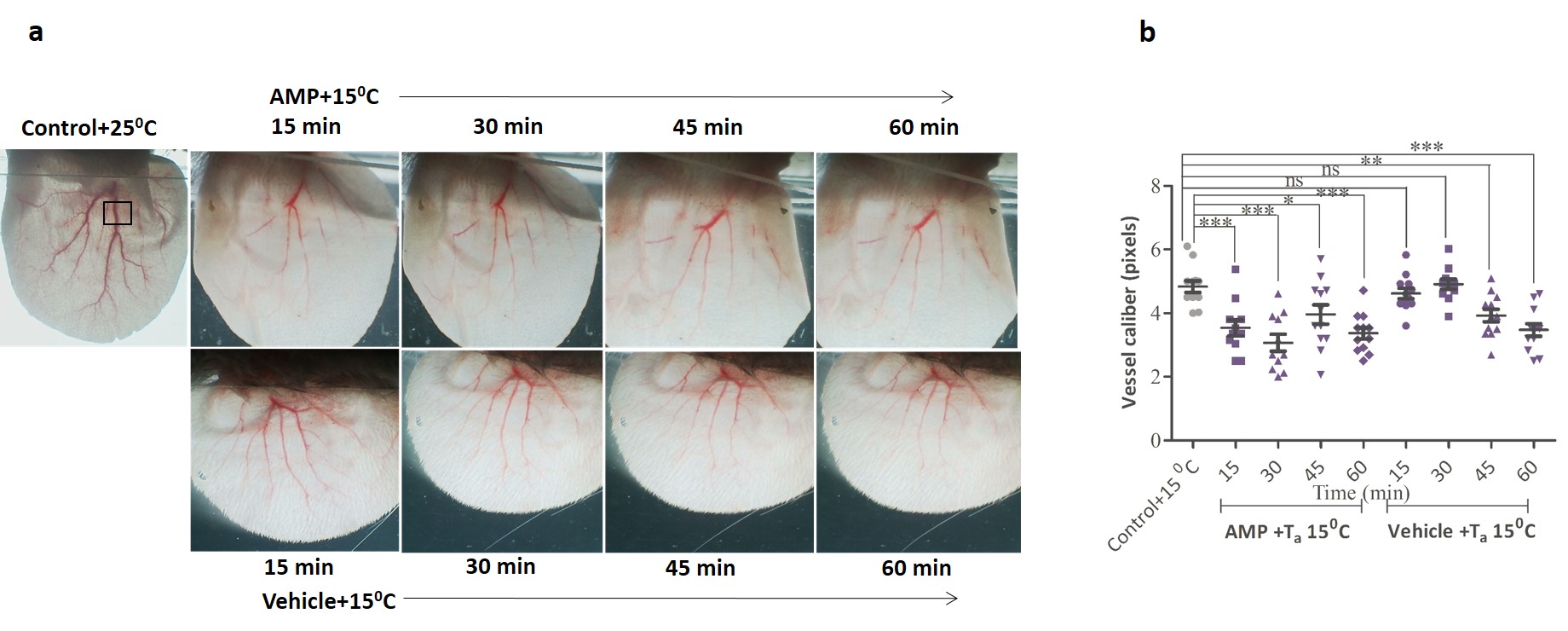
**

**Supplementary Figure 3: AMP induced HMS reduces peripheral blood circulation.** BALB/c male mice were administered with AMP (0.5 mg/g b.w.) and placed at a T_a_. At different time intervals the blood circulation in the ear of mice was photographed under a Stereozoom microscope for visualizing the blood micro circulation. (**a**) Representative image of ear of an untreated control mice, (**b-e**) images of ear of mice taken at 15, 30, 45 or 60 minutes after AMP treatment, placed at 15 °C. Magnification: 1.5×.


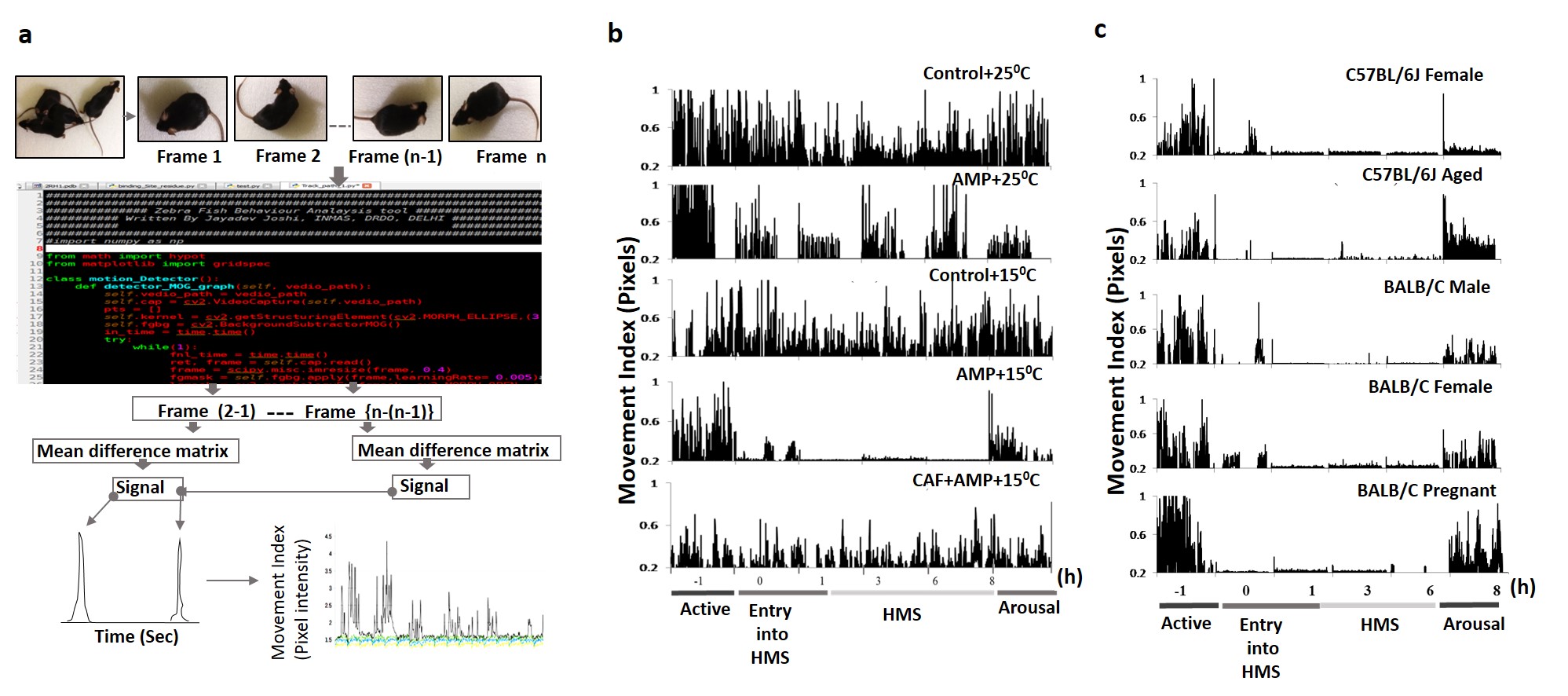


**Supplementary Figure 4: Effect of AMP induced hypometabolism on movement and activity of mice.** Mice were treated with AMP and placed at a T_a_ of 15°C. The cage activity/movement of mice was video graphed intermittently (every 1 hour) for a duration of 8 hours and processed using an in house developed algorithm. (**a**) The algorithm developed for processing the video and calculating the activity in terms of movement index (MI). Captured video was processed with the python and Open CV based script which subtracts the sequential frames and calculates the difference matrices which was further used to calculate the mean of difference matrix over time. This mean value of difference matrix was represented as a signal for movement. An MI of 1 represents a freely moving animal while a reduced value indicates a restricted or slow movement. (**b**) The MI was calculated for a total duration of 8 hours after indicated treatment. (**c**) The calculated MI in different mouse strains and sex. Caf represents caffeine.


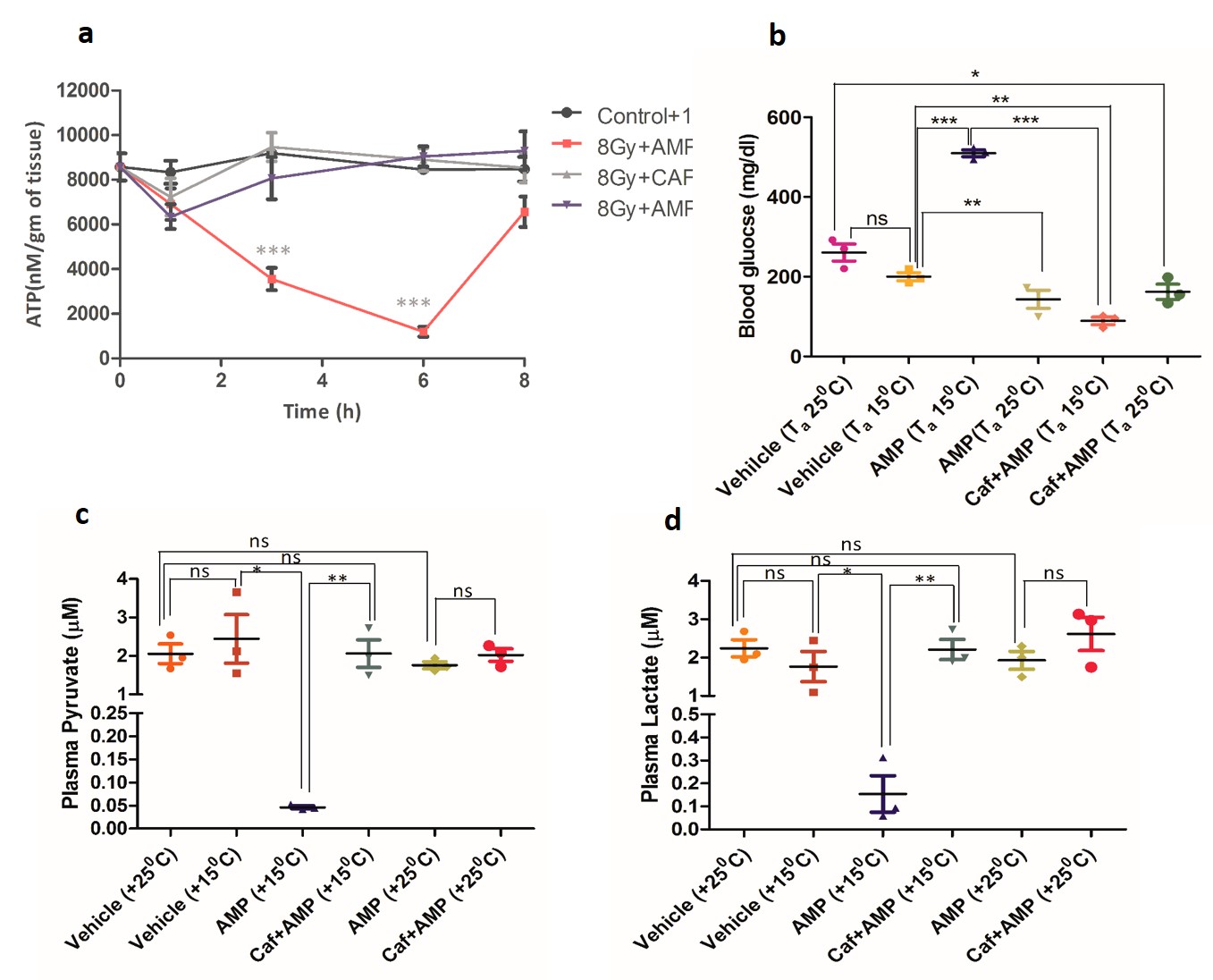


**Supplementary Figure 5: Effect of AMP induced HMS on tissue ATP levels.** Mice were treated with AMP and placed at a T_a_ of 15°C. After different time intervals mice were sacrificed and total ATP levels were quantified in liver tissue (two way ANOVA after Boneferoni post-test: F=8.305 and P<0.0001 for interaction, F=5.406 and P=0.0008 for time, F=30.37 and P<0.0001 for treatment, ***p<0.001 for AMP+15^0^C when compare to control+25^0^). **AMP induced HMS modulates glucose metabolism**. Changes in blood glucose (**b**), pyruvate (**c**) and lactate (**d**) observed 6 hours after different treatments. Each value is a mean ± SEM (n=3-6 mice/group) and comparisons were made between indicated groups. *p<0.05, **p<0.01, ***p<0.001. Caf: represents caffeine. Each value represents a mean ± SEM. Caf represents caffeine.


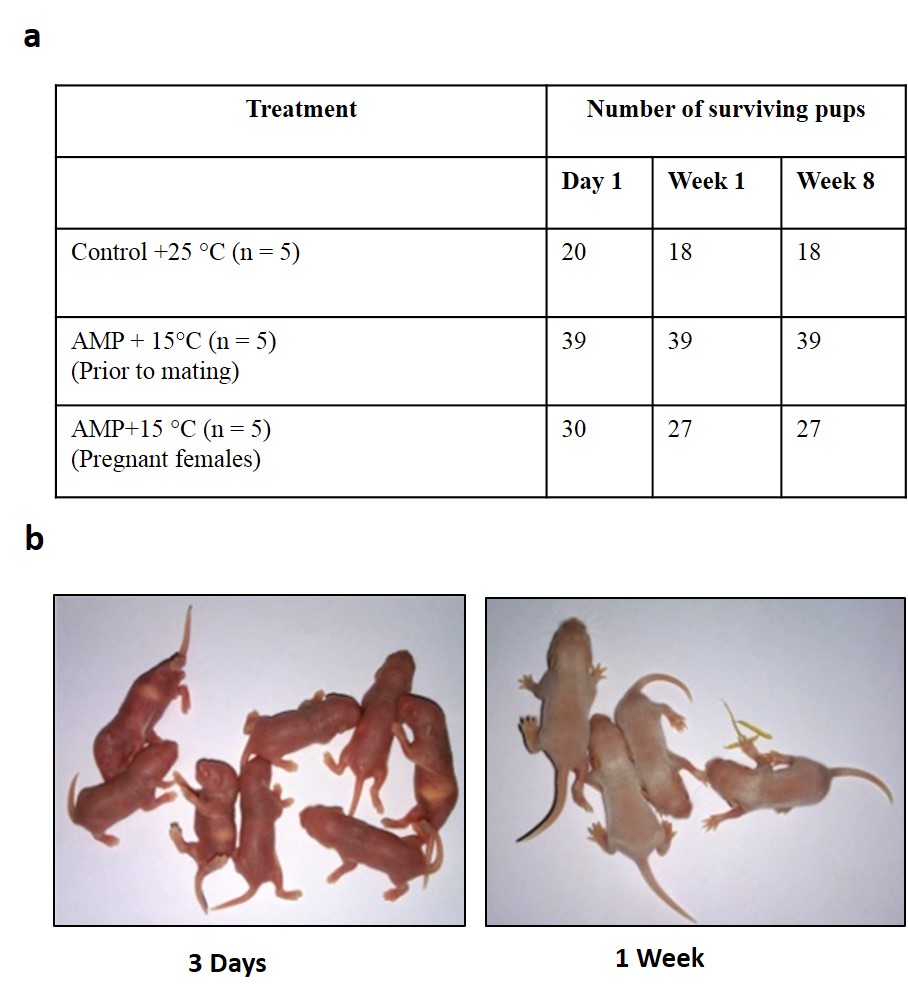


**Supplementary Figure 6: Effect of AMP induced HMS on reproductive capacity and foetal development**. HMS was induced in either pregnant female BALB/c mice or normal female mice. Thereafter the female mice underwent HMS for 6 hours and were allowed to mate with the male mice. The number, normal development and survival of pups delivered were observed for up to 8 weeks. (**a**) Representative images of pups delivered after 3 and 7 days. (**b**) Survival of pups delivered after different treatments. The numbers in parenthesis are the total number of animals used in the study.

**
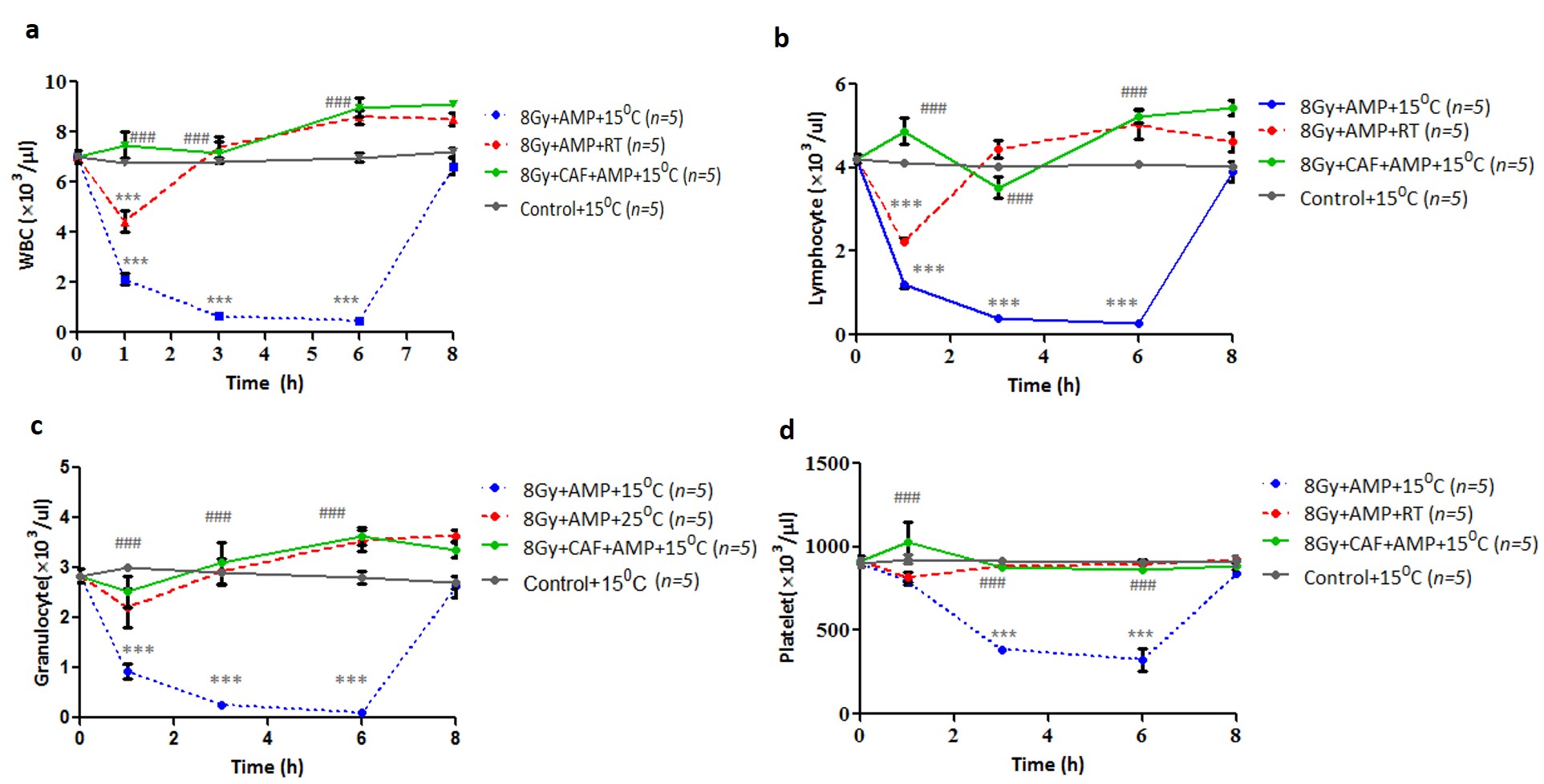
**

**Supplementary Figure 7: AMP reversibly induces disappearance of lymphocytes from peripheral blood**. (**a**) Disappearance of WBCs (two way ANOVA after Boneferoni post-test: F=38.66 and P<0.0001 for interaction, F=58.51 and P<0.0001 for time, F=244.3 and P=0.0001 for treatment. ***p<0.001, for 8Gy+AMP+15^0^C and 8Gy+AMP+25^0^C when compared to control+150C. ###p<0.0001 for 8Gy+AMP+150C when compared to CAF+8Gy+AMP+15^0^C). (**b**) Disappearance of lymphocyte (two way ANOVA after Boneferoni post-test: F=42.24 and P<0.0001 for interaction, F=53.46 and P<0.0001 for time, F=380.1 and P=0.0001 for treatment.***p<0.001, for 8Gy+AMP+15^0^C and 8Gy+AMP+25^0^C when compared to control+15^0^C. ###p<0.0001 for 8Gy+AMP+15^0^C when compared to CAF+8Gy+AMP+15^0^C). (**c**) Disappearance of granulocyte (two way ANOVA after Boneferoni post-test: F=14.57 and P<0.0001 for interaction, F=15.25 and P<0.0001 for time, F=73.69 and P=0.0001 for treatment. ***p<0.001 for 8Gy+AMP+15^0^C when compared to Control+15^0^C and ###p<0.001 for when 8Gy+AMP+15^0^C compared to 8Gy+CAF+AMP+15^0^C). (**d**) Disappearance of platelets (two way ANOVA after Boneferoni post-test: F=14.17 and P<0.0001 for interaction, F=18.21 and P<0.0001 for time, F=43.37 and P=0.0001 for treatment. ***p<0.001 for 8Gy+AMP+15^0^C when compared to Control+15^0^C and ###p<0.001 for when 8Gy+AMP+15^0^C compared to 8Gy+CAF+AMP+15^0^C). CAF represents caffeine.

**
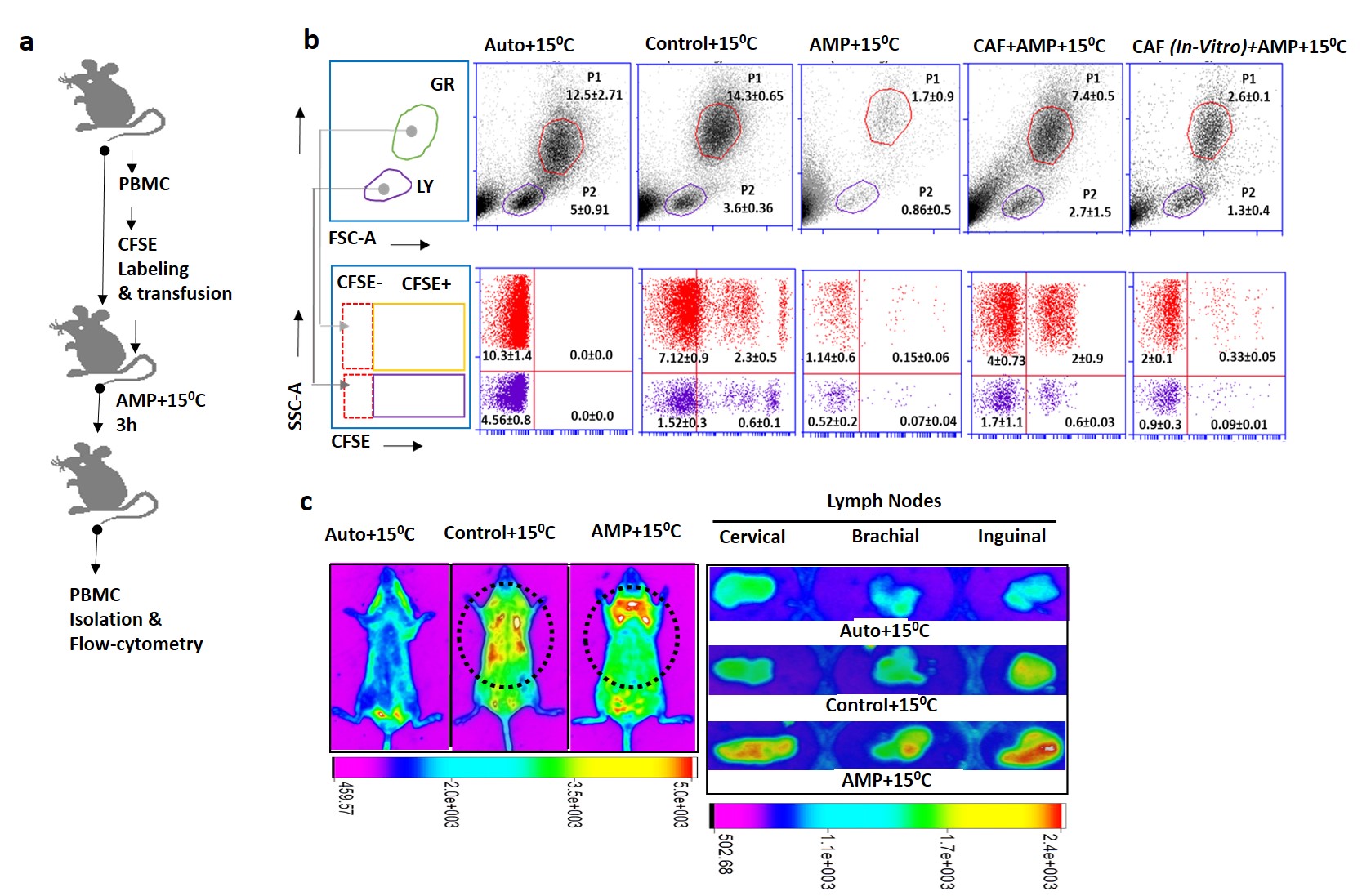
**

**Supplementary Figure 8: Effect of caffeine treatment on AMP induced disappearance of lymphocytes and granulocytes from peripheral blood.** (**a**) Schema of experimental set up for quantifying the AMP+15°C induced changes in lymphocytes and granulocytes in peripheral blood. CFSE labelled lymphocytes were administered into mice followed by AMP+15°C treatment. After 3 hours of treatment, peripheral blood was collected and changes in lymphocytes and granulocytes were quantified flow cytometrically. Lymphocyte and granulocyte populations were gated following staining with anti-Gr-1-PerCp (marker for granulocytes) and anti-CD45-APC (for lymphocytes) respectively. (**b**) Gating strategy used for detecting lymphocytes and granulocytes using their scatter properties (SSC vs FSC) or changes in fluorescence intensity (SSC vs CFSE) respectively and dot plots of the changes in CFSE fluorescence with different indicated treatments. (**c**) Tracking of CFSE labelled lymphocytes in vivo in AMP+15°C induced HMS. CFSE labelled lymphocytes (1×10^6^) were infused into B6 male mice followed by AMP+15°C treatment. After 3 hours of treatments, animals were anaesthetised and imaged non-invasively using optical imaging platform. For better contrast, an exposure time of 20 seconds was used and left panel images are the representative in vivo images of mice after different treatments and right panel is the representative image of ex vivo imaging of cervical, brachial and inguinal lymph nodes excised from the animals. Each value represents a mean ± SEM (n=6 animals; unpaired t test; comparisons were made between untreated control and AMP treated groups *p<0.05, **p<0.01, ***p<0.001). GR is granulocytes, LY lymphocytes. CAF represents caffeine.


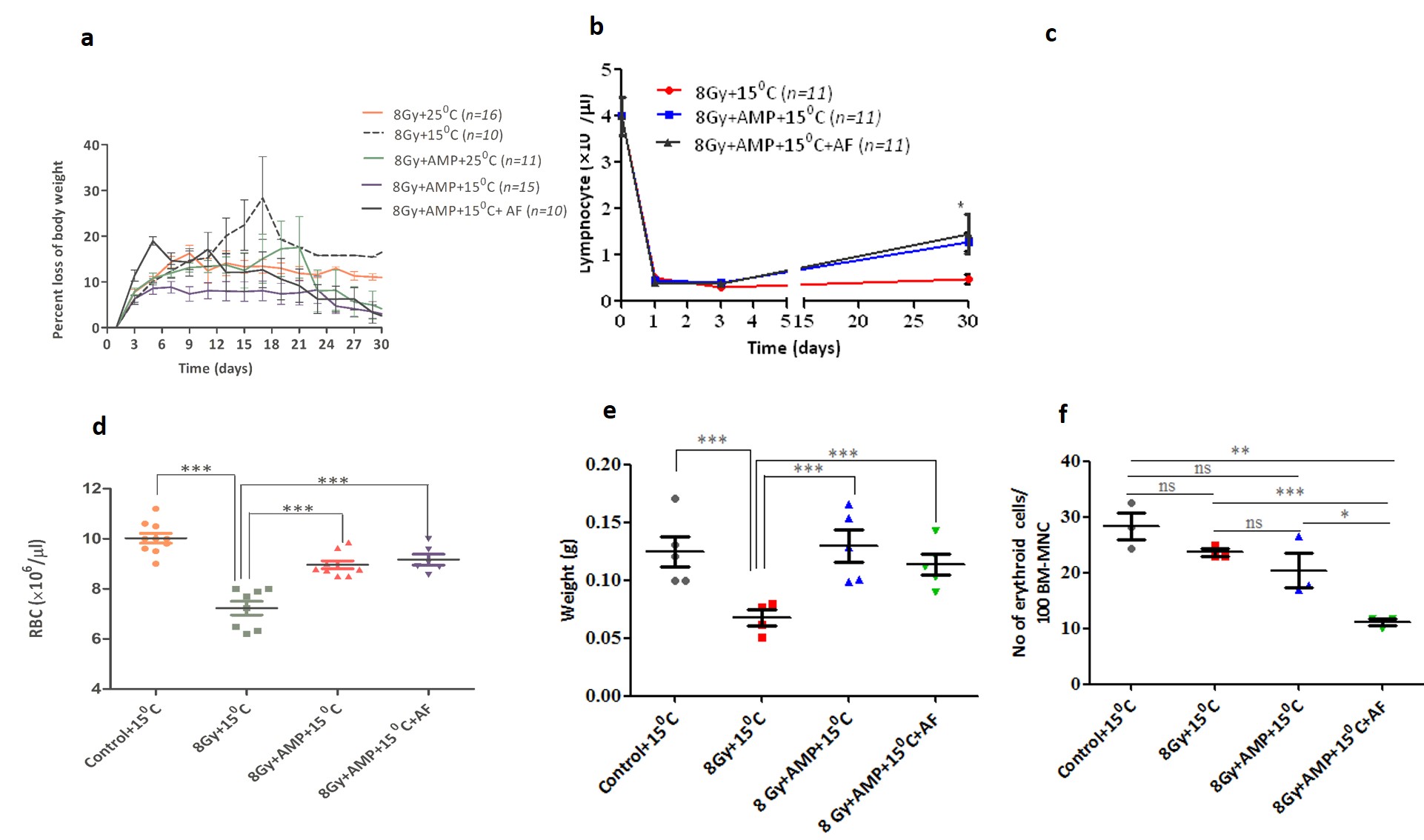


**Supplementary Figure 9: Radiomitigative effect of AMP induced HMS- Changes in body weight**. Daily changes in the body weight of mice represented as mean ± SEM. Comparisons were made between untreated control and AMP treated groups. **HMS induced by AMP+15°C improves post-irradiation hematopoietic recovery in mice.** Blood counts over time in B6 mice exposed to lethal doses of gamma radiation and treated with AMP+15°C. Changes in absolute number of (**a**) lymphocytes with time (two way ANOVA after Boneferoni post-test: F=1.187 and P=0.32 for interaction, F=157.4 and P<0.0001 for time, F=0.8193 and P=0.45 for treatment. *p<0.01 for 8Gy+AMP+15^0^C and 8Gy+AMP+15^0^C+AF when compare to 8Gy alone), (**b**) granulocytes with time (two way ANOVA after Boneferoni post-test: F=2.326 and P=0.039 for interaction, F=135.9 and P<0.0001 for time, F=3.649 and P=0.038 for treatment. ***p<0.001 for 8Gy+AMP+15^0^C+AF when compare to 8Gy alone). (**c**) RBC over the course of 30 post- irradiation days. (**d**) Changes in the spleen weight on 30th post-irradiation day after different treatments. (**e**) Representative images of longitudinal section of femur harvested 30 days after different treatment and stained with H&E. (**f**) Changes in the number of erythroid progenitors per 100 nucleated bone marrow cells. Each value is a mean ± SEM (n=4-6 mice/group) and comparisons were made between indicated groups. *p<0.05, **p<0.01, ***p<0.001. Scale bar=100µm. AF represents amifostine.


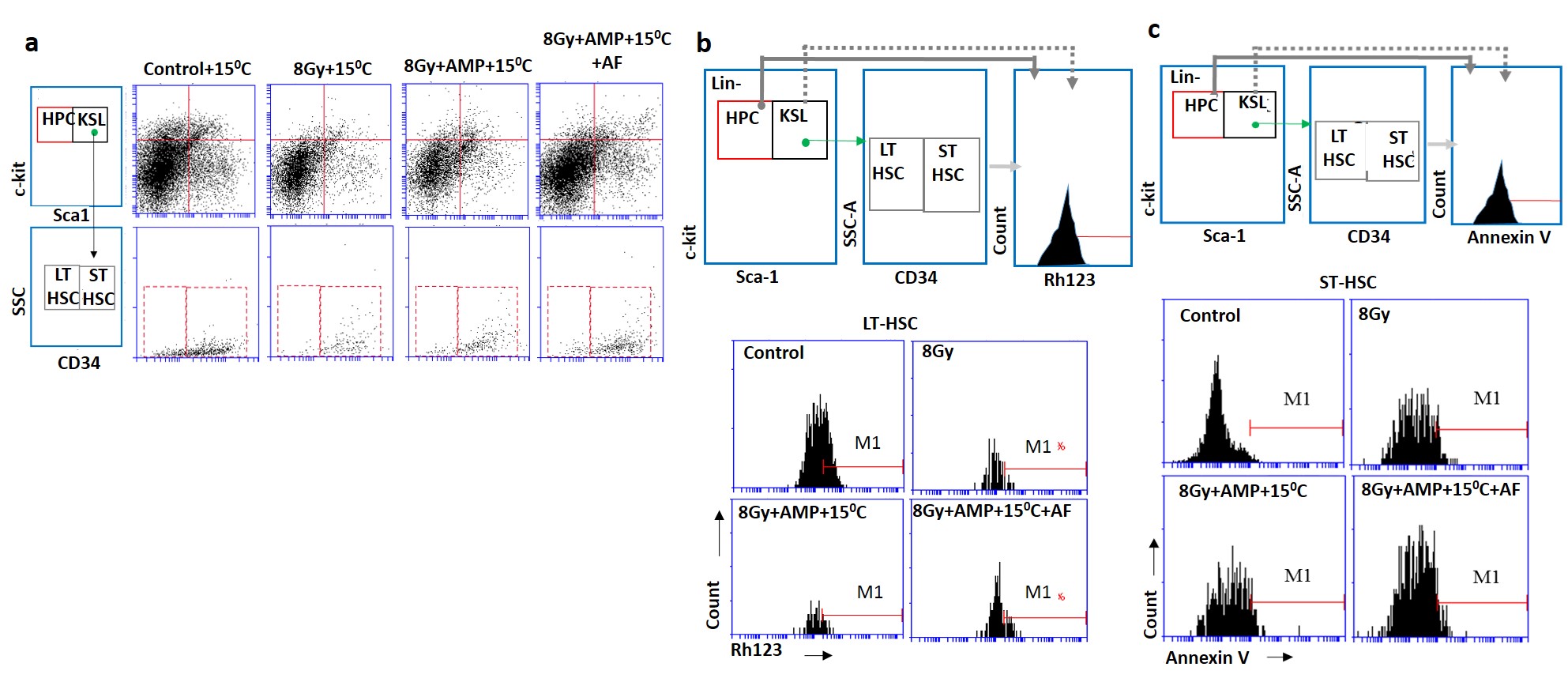


**Supplementary Figure 10: Gating strategy adopted for HSPCs enumeration, mitochondrial membrane potential and annexin V^+^ population.** (**a**) Representative gating strategy of flow cytometry analysis for multipotent progenitor (Lin^–^Sca1^–^c-Kit1^+^ cells), LSK cells (Lin^-^Sca1^+^c-Kit1^+^ cells), ST-HSCs (Lin^-^Sca1^+^c-Kit1^+^ CD34^+^ cells) and LT-HSCs (Lin^-^Sca1^+^c-Kit1^+^ CD34^-^ cells) in Lin^-^BMMNCs harvested 24 hours after different treatments. (**b**) Gating strategy used for identifying different stem and progenitor population and determination of MMP. (**c**) Gating strategy used for identifying stem and progenitor population and determination of annexin V^+^ (apoptotic fraction) in a representative (ST-HSCs) cell population. AF represents amifostine.


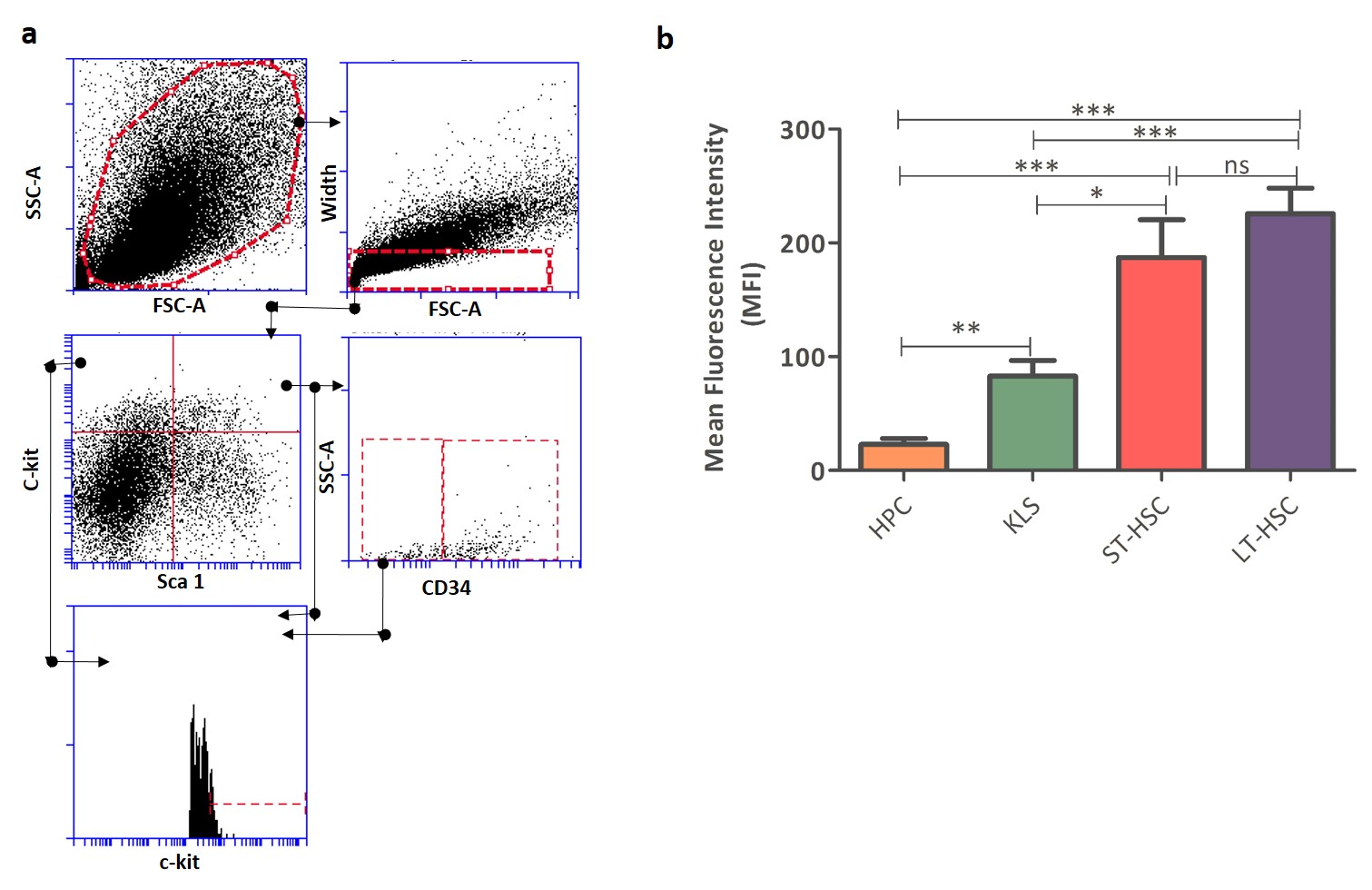


**Supplementary Figure 11: Cell surface c-Kit expression in different subsets of HSPCs.** (**a**) Gating strategy adopted for calculating surface expression of c-Kit expression in different subsets of HSPCs (mean fluorescent intensity (MFI). (**b**) Changes in the surface c-Kit expression in different subsets of HPSC during steady state haematopoiesis. Each value is a mean ± SEM (n=6 mice/group) and comparisons were made between indicated groups. *p<0.05, **p<0.01, ***p<0.001.


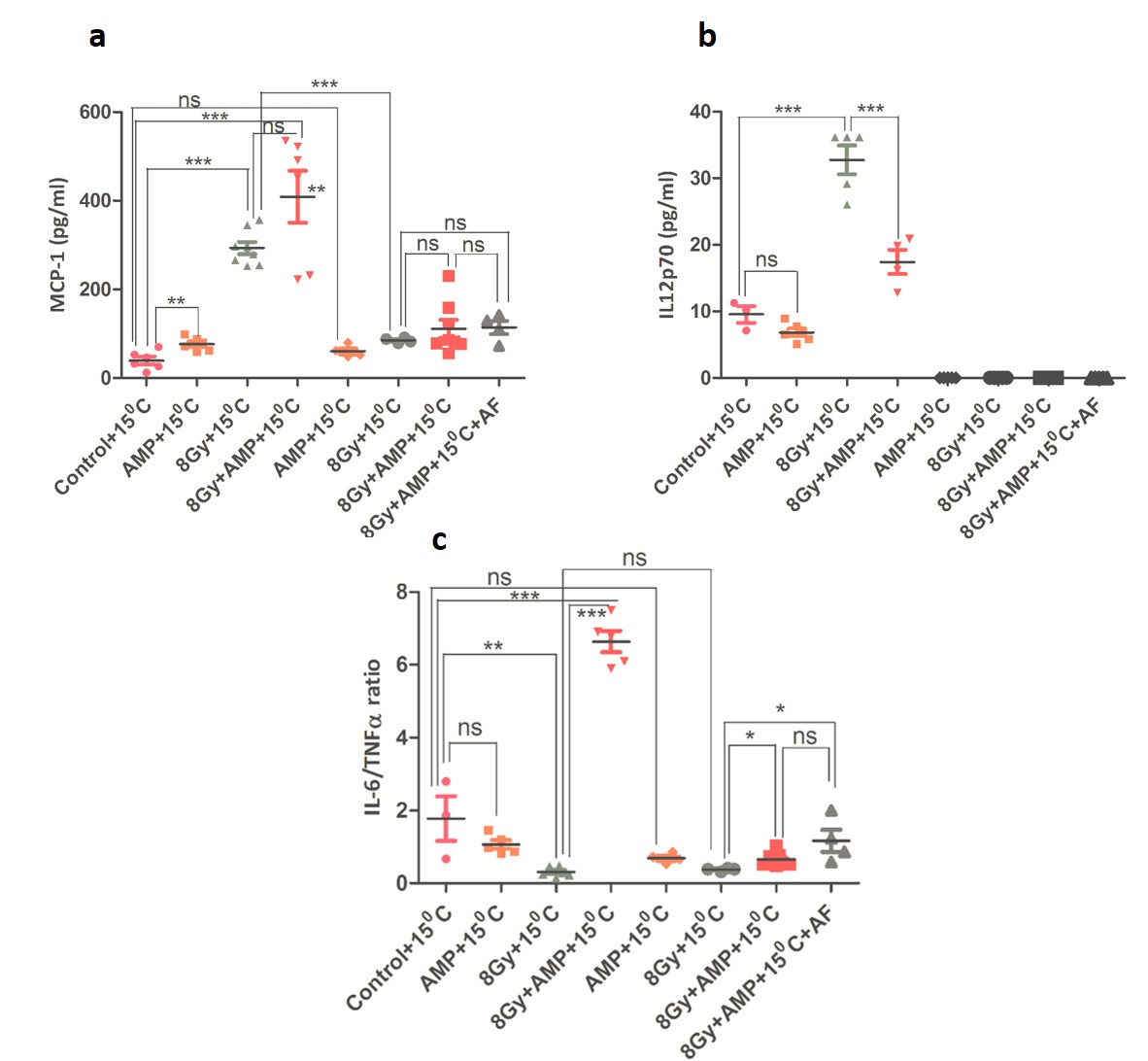


**Supplementary Figure 12: Changes in IL-12p70 and MCP levels in serum collected at different time points after varied treatments.** At 3 and 24 hours of different treatment, blood was collected, serum was harvested and levels of IL-12p70 (**a**) and MCP-1 (**b**) were measured flow cytometrically using cytometric bead array. (**c**) The ratio of anti-inflammatory to pro-inflammatory (IL-6/TNF-α) cytokines which represents the prevailing inflammatory status. Each value is a mean ± SEM (n=6 mice/group) and comparisons were made between indicated groups. *p<0.05, **p<0.01, ***p<0.001. A number of data points are outside the axis limits. AF represents amifostine.

**Supplementary Video: A time laps recording of mice in deep HMS and resuscitation.** Irradiated mice were injected with AMP (0.5 mg/g b.w.) and placed at a T_a_ of 15°C.
